# Steroid Androgen Exposure during Development Has No Effect on Reproductive Physiology of *Biomphalaria glabrata*

**DOI:** 10.1371/journal.pone.0159852

**Published:** 2016-07-22

**Authors:** Satwant Kaur, Alice Baynes, Anne E. Lockyer, Edwin J. Routledge, Catherine S. Jones, Leslie R. Noble, Susan Jobling

**Affiliations:** 1 Institute of Environment, Health and Societies, Brunel University London, Uxbridge, UB8 3PH, United Kingdom; 2 Institute of Biological and Environmental Sciences, School of Biological Sciences, University of Aberdeen, Aberdeen, AB24 2TZ, Scotland; University of Calgary, CANADA

## Abstract

Gastropod mollusks have been proposed as alternative models for male reproductive toxicity testing, due to similarities in their reproductive anatomy compared to mammals, together with evidence that endocrine disrupting chemicals can cause effects in some mollusks analogous to those seen in mammals. To test this hypothesis, we used the freshwater pulmonate snail, *Biomphalaria glabrata*, for which various genetic tools and a draft genome have recently become available, to investigate the effects of two steroid androgens on the development of mollusk secondary sexual organs. Here we present the results of exposures to two potent androgens, the vertebrate steroid; 5α-dihydrotestosterone (DHT) and the pharmaceutical anabolic steroid; 17α-methyltestosterone (MT), under continuous flow-through conditions throughout embryonic development and up to sexual maturity. Secondary sexual gland morphology, histopathology and differential gene expression analysis were used to determine whether steroid androgens stimulated or inhibited organ development. No significant differences between tissues from control and exposed snails were identified, suggesting that these androgens elicited no biologically detectable response normally associated with exposure to androgens in vertebrate model systems. Identifying no effect of androgens in this mollusk is significant, not only in the context of the suitability of mollusks as alternative model organisms for testing vertebrate androgen receptor agonists but also, if applicable to other similar mollusks, in terms of the likely impacts of androgens and anti-androgenic pollutants present in the aquatic environment.

## Introduction

Declining male reproductive health in humans [1,2] is of particular current concern, and has increased the need for animal experiments to investigate both treatments and causes of male reproductive disorders and diseases. The possibility that these disorders may, in part, be caused by exposure to environmental pollutants that disrupt the male reproductive endocrine system [3,4] has led to changes in legislation, resulting in an increased need for animal testing to determine reproductive toxicity. The Hershberger assay [5], originally developed to identify the drivers (hormones) of male sexual development, is a gold standard test used in reproductive toxicity testing as well as the development of pharmaceuticals designed to treat male reproductive disorders and hormone-dependent cancers [6]. This assay employs castrated peri-pubertal male rats to assess effects of chemical or pharmaceutical exposure on weight, morphology and histopathology of reproductive organs and tissues and is, therefore, both ethically undesirable and costly. To replace the rodents used in reproductive pharmacology and toxicological testing with small invertebrate organisms is desirable, to reduce both the use of rodents and the high costs of such tests. This approach has been successful in, for example, research into Parkinson's disease, axon regeneration and Alzheimer's disease through the use of the worm, *Caenorhabditis elegans*, and the fruitfly, *Drosophila melanogaster* [7–10]. Neither of these organisms, however, have a reproductive anatomy similar to mammals and so would not be suitable alternatives with which to investigate the male reproductive system. Few other invertebrates have been extensively investigated as potential surrogates of male (or female) reproductive function, for either biomedical or chemical safety testing, however mollusks have been proposed as potential surrogates for androgen testing, since they have a reproductive anatomy that is, at least superficially, similar to that of vertebrates. Like mammals, the snail’s male reproductive tract extends from the testis (or ovotestis in hermaphrodite snails), has seminal vesicles and a prostate gland, and terminates in a penile glandular complex (around the preputium) containing sebaceous glands and a penis.

Evidence from toxicological studies also indicate possible commonalities in the reproductive development and reproductive responses of some mollusk species with those seen in mammals [11–13]. For example, developmental exposure to the synthetic androgen, 17α-methyltestosterone (MT) caused hyperplasia in the epithelial tissue of reproductive glands and inhibition of spermatogenesis in male *Marisa cornuarietis* (a freshwater prosobranch/gonochoristic gastropod mollusk) [14] while female adult *M*. *cornuarietis* developed imposex (imposition of penis growth in females) after 150 days of MT exposure [15]. Adult pond snails, *Lymnaea stagnalis* (a pulmonate/hermaphrodite gastropod), demonstrated histological damage to the albumen and prostate glands after 8 weeks of exposure to 100 ng/L of MT [16]. Studies with gastropods have also shown exposure to vertebrate androgens (e.g. testosterone), anti-androgens (e.g. cyproterone-acetate) or estrogens could modulate free or esterified testosterone concentrations within the exposed snails [17,18], suggesting exogenous exposures to vertebrate-type endocrine active substances may disrupt androgen homeostasis in mollusks [18]. Moreover, exposure of males of different gastropod species (*M*. *cornuarietis*, *Nucella lapillus* and *Hinia reticulate*a) to the fungicide vinclozolin, shown to have anti-androgenic properties in mammals, were reported to cause a reduction in the length of the penis and penis sheath [14], further supporting the suggestion that mammalian-like mechanisms through which androgens and anti-androgens could act may be present in mollusks. Based on the above literature it was expected that androgen exposure might stimulate the growth of male specific tissues (e.g. prostate) and cells (e.g. sperm), and perhaps inhibit the growth of female specific tissues (e.g. albumen gland) and cells (e.g. oocytes).

Although their function has yet to be fully elucidated, putative vertebrate-like male hormones such as testosterone and dihydrotestosterone; as well as steroidogenic androgen- and estrogen-producing enzymes, such as aromatase and 5α-reductase; have been found in the gonads and digestive tract of several invertebrate groups [19–23], particularly mollusks and annelids. Moreover, the genes encoding for 5α-reductase have been sequenced in several mollusk species (e.g. *Crassostrea gigas* (ECK36980), *Lottia gigantea* (Pid: 194080)), perhaps suggesting that similar mechanisms exist in invertebrates. Prior to the commencement of the research presented in this manuscript, the presence of a ‘classical’ androgen receptor (AR) in mollusks had been inferred [24,25], but this has remained an area of controversy [26], since homologues have not been identified despite investigations specifically searching for the gene [27]. Recently, systematic searches of mollusk genomes have failed to identify an androgen receptor [28,29] homolog suggesting that the reported effects of androgens in mollusks must be attributed to a different mechanism, either using different nuclear receptors or potentially through non-genomic membrane-bound receptors. The aim of this study was to test the hypothesis that genes, proteins and physiological processes that are common to both mollusks and mammals underlie the response of the male reproductive system to androgens.

The tropical freshwater pulmonate snail, *B*. *glabrata*, was selected as a test species. In comparison to other gastropods, it has a relatively well-documented physiology and had been widely studied, including a recent preliminary genome sequence [30], due to its medical importance as the intermediate host for the human parasite *Schistosoma mansoni*. It is also easily bred in the laboratory with a short generation time, thus making it an attractive test model for mechanistic research and *in vivo* testing. *B*. *glabrata* were exposed during development to two potent androgens; the natural vertebrate steroid, dihydrotestosterone (DHT) and the anabolic steroid methyltestosterone (MT), after which reproductive tissues and organs were assessed for stimulatory (e.g. male tissue reproductive growth or advanced spermatogenesis) or possible disruptive (e.g. to female reproductive tissues or oocyte development) effects using morphological, histopathological and differential gene expression analyses, to test the hypothesis that androgens would elicit similar effects in mollusks to that seen in mammals.

## Materials and Methods

### Ethics statement

Ethical approval of from the Brunel University London Animal Welfare Ethics Review Board (AWERB) and the UK Home Office was not required for this study as gastropod mollusks, such as *B*. *glabrata*, are not protected by the UK Animal [Scientific Procedures] Act 1986. However, as the work was *in vivo* in nature all efforts were made to optimize husbandry conditions and minimize possible suffering. This manuscript was prepared with ‘Animal Research: Reporting *In Vivo* Experiments’ (ARRIVE) guidelines [31] in mind.

### Test organism

The hermaphrodite planorbid freshwater snail *B*. *glabrata* (BB02 strain; obtained originally from The Natural History Museum, London), were maintained in a recirculation system in glass aquaria supplied with de-chlorinated tap water. Snails were fed *ad libitum* on alternate days with Tetramin fish flakes (roughly ~10% of their body mass). A light regime of 16: 8 L: D, incorporating 20 min dawn/dusk transition periods, was maintained over the course of the experiment. Tank water conditions were monitored throughout the study; temperature (27 ± 1°C), pH (8 ± 0.21), dissolved oxygen (6 ± 2 mg/L) and ammonia (0 mg/l), nitrate (0mg/l) and nitrite (<40 mg/l).

### Test compounds

Concentrated stock solutions (5 g/L) of androgens 17α-methyltestosterone (MT) (CAS No. 564-35-2) and 5x-dihydrotestosterone (DHT) (CAS No. 521-18-6) (Sigma Aldrich Co. Ltd, UK) were prepared in HPLC grade N, N-dimethylformamide (DMF) 99% stored at 4°C. Dosing stocks were prepared twice a week in double distilled water. Total DMF solvent concentration in all dosing stock bottles was maintained at 6%.

### Experimental design

Two separate 30-day experiments were conducted; one for DHT and one for MT. 40 snails per treatment (20 per replicate tank) were exposed to each of the test chemicals throughout embryonic and early development until sexual maturation (age at which egg laying begins). Each experiment consisted of six treatments (tested in duplicate): dilution water control (DWC), solvent control 0.006% DMF (SC), plus MT or DHT at nominal concentrations of 62.5 ng/L, 125 ng/L, 250 ng/L, and 500 ng/L. Test concentrations were chosen to be in the range of androgen concentrations previously reported to affect gastropod mollusks and fish [14,32–34].

### Egg collection and exposure of snails

Four *B*. *glabrata* egg masses (~30–50 eggs/mass) per treatment (i.e. DWC, SC, 62.5, 125, 250 and 500 ng/L MT or DHT (total egg masses n = 24)) were exposed in individual wells of a six-well plate (Nunc), from the day they were laid throughout embryonic development. Half of the appropriate treatment water was replaced (3 mL total volume) every 24 hours. Egg masses were inspected daily for development and hatching. 7 days post-oviposition, 40 synchronously hatched snails were selected for continued exposure for each treatment. Duplicate groups of 20 snails per treatment (total snails n = 240) were exposed under continuous dosing (flow-through) conditions in 3.5L glass tanks for 23 days until point of sexual maturity, (i.e. age at which egg laying is generally first observed). Water and androgen dosing stocks were delivered into glass mixing chambers at nominal concentrations in the exposure tanks of 62.5, 125, 250 and 500 ng/L (MT or DHT) using medical grade silicon tubing (Watson Marlow, UK). Treatments, (except DWC) contained DMF at 0.006% v/v (below the suggested maximum solvent concentration of 0.01% v/v OECD limit for aquatic vertebrates OECD 2000 and gastropods OECD 2010), and were freshly prepared and replaced every 72 hours. Tank water was gently aerated to maintain dissolved oxygen levels.

### Tank water analyses

The target chemical (MT or DHT) was extracted from weekly-collected water samples (250 mL) from day 0 (before egg-masses were exposed) by solid phase extraction (SPE) using C18 SPE cartridges (Sep-pak Plus, Waters Ltd. UK) as per the manufacturer’s instructions. Sample extracts (double distilled water ‘blanks’, MT/DHT ‘spiked’ double distilled water and tank water samples) were eluted with analytical grade methanol (Fisher scientific, UK) and evaporated under a stream of nitrogen. Dried extracts were re-suspended in analytical grade ethanol (Hayman, UK) and stored at 4°C until further analysis using a recombinant Yeast androgen screen (YAS) [35]. Briefly, tank water (and blanks and spiked water) extracts were serially diluted in the assay plate and assayed against a DHT or MT standard curve (DHT starting concentration 1 x 10^−6^ M and MT 6.6 x 10^−7^ M) thus allowing tank water concentrations (expressed as ng DHT equivalent/L or ng MT equivalent/L) to be determined (taking in account the concentration factor of the SPE method).

### Tissue sampling

After 30 days exposure, snails were weighed (g) and shell diameter measured (mm) before killing by quick decapitation with a scalpel. The shell was then removed and the body re-weighed, then fixed in RNAlater and stored at 4°C. Subsequently the albumen gland (AG) and the ovotestis (OT) (S1 Fig) were dissected from snails >5 mm in shell diameter (snails <5 mm shell diameter were too small to be accurately dissected and further histopathology or molecular biology was not conducted), weighed (whole) and split for molecular analysis (stored in RNAlater at 4°C) and histological analysis (treated with Bouin’s solution (Sigma-Aldrich) and stored in 70% alcohol). The GC (containing the prostate, membrane and mucous glands) was also dissected out and weighed, however due to its complex nature (i.e. 3 gland types intertwining into each other) these were only sampled for histopathology. RNAlater was used as the primary fixative for the whole bodies, so that tissues could be assessed for both molecular and histopathology endpoints. This resulted in some differences in staining compared to primary fixation with Bouin’s solution (See S2 Fig for comparison).

### Histopathology processing and analyses

Fixed tissues were serially dehydrated and wax embedded using standard methods. Sections (5 μm) were stained with Mayer’s haematoxylin and 1% aqueous eosin before being examined for cell type and structure. All individuals were assessed and photographed (Q Imaging Micropublisher 5.0RTV, Q Capture Pro 5.1 software).

### Morphometric analysis of glandular complex

For each snail, the whole dissected GC was placed on a grid (cm^2^) to assess cross-sectional area. Images were captured (Leica MZ FLIII binocular dissecting microscope with a Leica DC300F digital camera) and analysed with ImageJ (http://rsb.info.nih.gov).

### RNA extraction and cDNA synthesis

Total RNA was extracted from individual AG or OT using the SV total RNA isolation system (Promega UK Ltd, Southampton, UK) according to the manufacturer’s protocol. This kit includes DNAse treatment to eliminate genomic DNA contamination. For generating the SSH libraries, an equal quantity of RNA from 10 individuals was pooled for cDNA synthesis and subsequent amplification used 1 μg pooled total RNA with the SMARTer^™^ PCR cDNA synthesis kit (Clontech-Takara Bio, Europe, France) according to the manufacturer’s instructions. For testing differential expression of identified transcripts on individual snails, cDNA synthesis was carried out using 300 ng total RNA (from above) from each individual using Superscript III first strand synthesis system (Life Technologies) priming with random hexamers and finishing with an RNAse H digestion according to the manufacturer’s protocol.

### Suppression subtractive hybridization and library screening

Four subtracted libraries for MT (125 ng/L) exposed snails versus solvent control and vice versa for both the AG and OT were made using the PCR Select kit (Clontech-Takara Bio, Europe, France) as described in the manufacturers’ instructions. To minimize sampling effects, 6 primary amplification PCRs followed by a secondary PCR from each primary PCR were performed and the 6 secondary PCRs pooled for each library construction. 3 μL of each pool of secondary PCRs was ligated into pGem-T easy vector (Promega) and used to transform JM109 cells according to the manufacturer’s instructions. Each library was plated out onto 10 LB agar plates and 2 x 96 white colonies for each were cultured in 0.5 mL LB at 37°C overnight. Duplicate filters were made from these for each library using Bio-dot apparatus (Biorad). Briefly Biodyne B membrane (PALL) was soaked in 2 x SSC prior to loading in the press. Then 50 μL 2 x SSC with dilute bromophenol blue was sucked through followed by 20 μL LB culture, then denatured with 50 μL denaturing solution (1.5 M NaCl, 0.5 M NaOH), neutralized with 50 μL of 1.5 M NaCl, 0.5 M Tris pH 7.5 solution for 2 mins, and rinsed in 50 μL 2xSSC. The filters were air dried and stored at RT. For each pair of duplicate filters, one was probed with 10–15 μL of cleaned secondary PCR, previously digested with the restriction enzymes, *Sma*I and *Rsa*I (Boerhinger Mannheim) to remove adapter sequences from the library from which it was made, and one with a probe from the reciprocal subtracted library prepared in the same way. For example, the MT-exposed AG-specific library filters were probed with the MT-exposed AG library, as well as the control AG library. Hybridizations were performed overnight in hybridization bags at 42°C using digoxigenin labelled probe (DIG High Prime DNA labelling detection starter kit II, Roche). Differentially expressed transcripts were identified as those that were clearly present on the filter probed with the corresponding library while completely absent from the reverse subtracted library.

### Sequence analysis

All identified differentially expressed fragments were sequenced using BigDye terminator cycle sequencing kit (Life Technologies) and run on an ABI 377 (Sequencing Facility, Wolfson Wellcome Biomedical Laboratory, The Natural History Museum, UK). The sequence traces were examined using Geneious (http://www.geneious.com) and all vector and adaptor sequences removed. Cluster analyses were performed using Seqtools (8.4ver) (http://www.seqtools.dk/). Unique sequences were compared to GenBANK using BlastN, BlastX and tBlastX using Blast2go (http://www.blast2go.com) and submitted to GenBank (Accession Numbers JZ875074-JZ875078).

### Quantitative PCR (qPCR)

qPCRs contained Fast SYBR^®^ Green Master Mix according to the manufacturers protocol (Applied Biosystems), 10 pmol of each primer (designed from the sequenced fragments using PRIMER3 (version 0.4.0) [http://bioinfo.ut.ee/primer3-0.4.0/] (Table 1)) and 1 μL of 1/5 dilution of cDNA synthesized from albumen gland from individual snails (see above). PCR cycling conditions were: 50°C for 2 min, 95°C for 10 min, then 40 cycles of 95°C for 30 sec, 57°C for 1 min, using the CFX96 Real-time PCR Detection system (Biorad). A dissociation curve was generated in each case to check that only a single band was amplified. The qPCRs were performed in triplicate for each individual snail and normalized to 18S (primers from Hertel *et al*. 2005 [36]). The results were analyzed using qGene [37] taking into account amplification efficiency, which was calculated using a dilution series for each primer pair.

**Table 1:** Primers used in the study.

Name	Forward 5’-3’	Reverse 5’-3’
18S	CGCCCGTCGCTACTATCG	ACGCCAGACCGAGACCAA
MTA23	AAACAAATCTCTCTCACACAGGAC	CGACACAAGTGGAAACGAAG
MTA34	CCCAAGCAGCCAGAAGCCAG	GCGACAAACTCATCCAGCCAGA
MTA18	AAGCAAACACGCTAAACACG	GTATCCAAATAACCGAGGAACAC
MTA21	AATCAATGCTCCACCTACGG	AATGTCCTGGCGGTCAATAC
MTA41	TGCGTCTGCTACTGGCTATG	GGACTTGGTTCTCAGGATTGG

### Statistical Analysis

Differences in shell diameter, total weight, normalized gland complex (GC) area, normalized albumen gland weight and normalized ovotestis weight were assessed using IBM SPSS (version 20). Shapiro-Wilk Test and normal Q-Q plots were used to assess data (shell diameter etc.) for normal distribution. Levene's Test was used to assess the equality of variances for different groups being compared (i.e. comparisons between replicates of the same treatment; T-test for comparisons between different treatments; ANOVA). For both the Shapiro-Wilk Test and Levene's Test α was set at 0.05, above which groups were considered to be normally distributed/to have equal variance. Prior to combining data from replicate tanks (from the same treatment), differences between replicate tanks were analyzed with either Student’s T-test or Welch’s T-test (depending on equal or un-equal variance). If no significant differences between replicates were found using T-tests, then data from the replicate tanks were combined into ‘treatments’ and further analyses were conducted using analysis of variance (ANOVA), if P≤ 0.05 for the ANOVA then post hoc analysis using Fisher’s least significant difference (LSD) were used to compare the treatment with the respective solvent controls. If T-tests found replicate tanks within the same treatment to be significantly different from each other, data sets could not be combined and comparisons by treatment could not be conducted. In these cases Tukey's HSD (honest significant difference) test were used to compare data (e.g. shell diameter, GC etc.) from all of the tanks individually to assess if the sub-sets produced would fall into treatment types, e.g. un-dosed (DWC, SC) vs. high dosed (250 ng/L, 500 ng/L), to look for any dose dependent groupings or trends. Statistical significance was considered at α ≤ 0.05 for all comparisons.

## Results

### Exposure analyses: confirmation of experimental tank water concentrations

**Fig 1:**
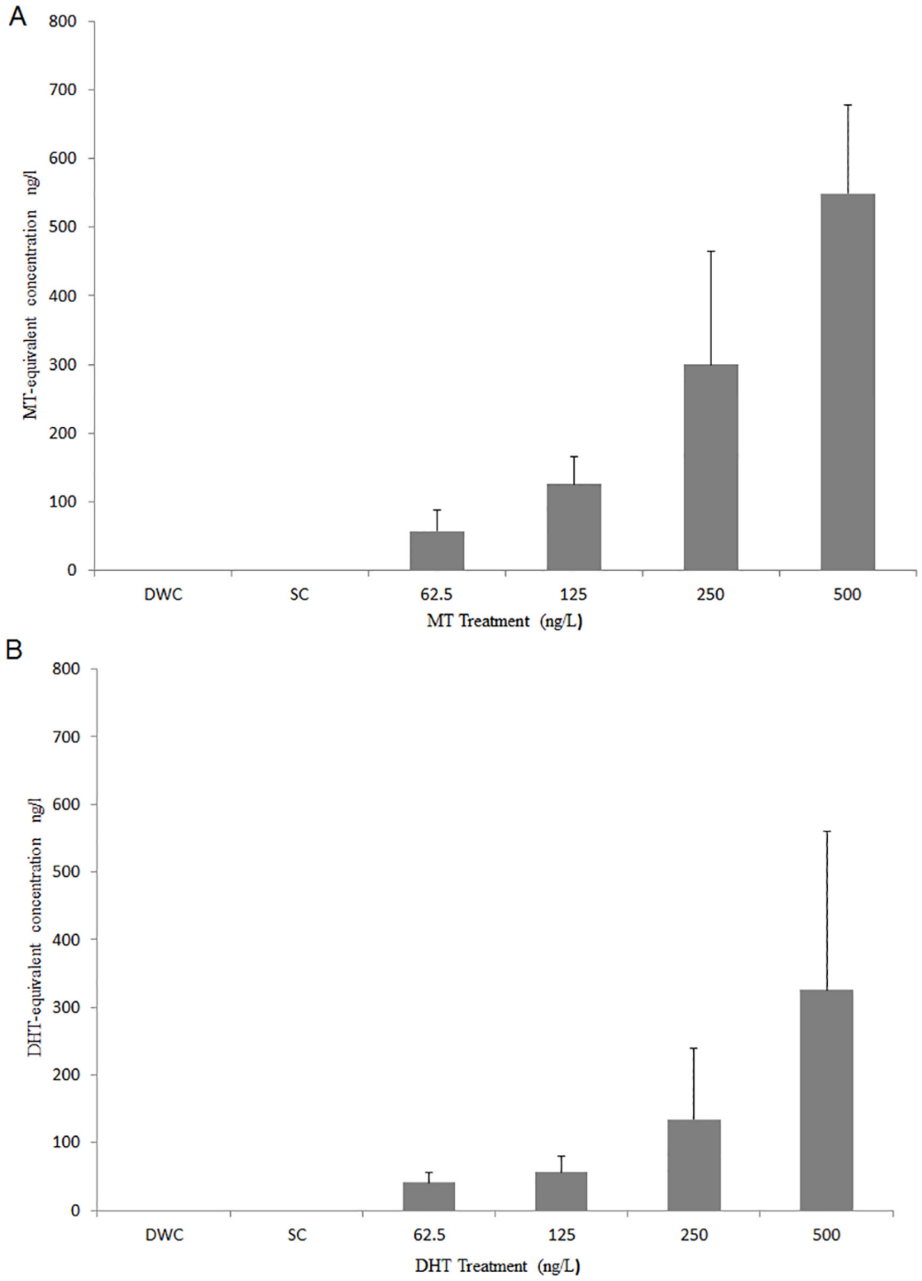
Average tank water concentration of methyltestosterone (A) or dihydrotestosterone (B) throughout *B*. *glabrata* 30-day exposure. 250 mL tank water samples were taken weekly (days 0, 7, 14, 21, and 28) and concentrated using C18 Solid Phase Extraction (SPE). Extracts were analyzed using YAS [35] against MT or DHT standard curves, as appropriate, to provide MT-equivalent or DHT-equivalent concentrations (tank water sample extract values calculated from the linear part of the standard curve(s)). All tank water extracts tested in duplicate. Treatments:—Dilution Water Control (DWC), Solvent Control (SC) and 62.5, 125, 250 and 500 ng/L of either MT or DHT. Mean calculated from all 5 time points. Error bars are standard deviation.

Analysis of biological activity (YAS assay) in water from the exposure tanks dosed with MT and DHT showed that snails were exposed to the test substances in the expected range of concentrations. No androgenic activity was detected in either the dilution water control (DWC), solvent control (SC) or extraction ‘blanks’ at any time, and no significant difference was found between concentrations in replicate tanks. Solid phase extraction efficiencies calculated from pure water spiked samples, were 93 ± 37.7% for DHT and 71.13 ± 21.1% for MT. Overall the actual tank concentrations (as measured by the YAS assay) were between 80 and 110% of nominal concentrations for MT and 65% to 70% of nominal concentration for DHT and increased in a dose-dependent manner (Fig 1A and 1B).

### Biological effects of exposure: mortality, growth and morphology

#### Snail survival and growth (shell diameter, weight)

**Fig 2:**
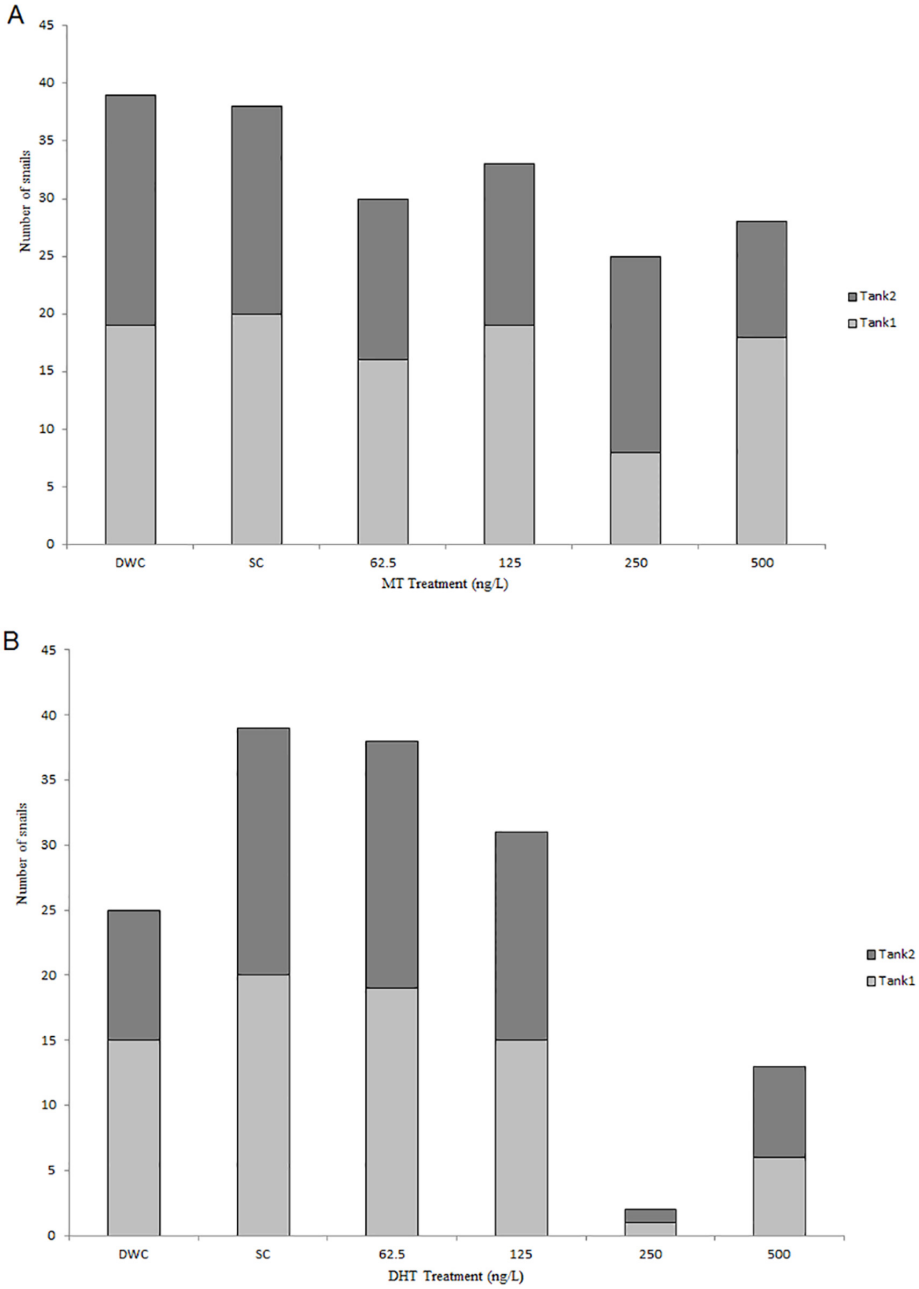
Number of *B*. *glabrata* per tank after 30-day exposure to methyltestosterone (A) or dihydrotestosterone (B). At the start of the experiment 20 hatchling snails were placed into each replicate tank (two tanks per treatment). Stacked bars represent the number of snails surviving in replicate tanks to the end of the 30-day developmental exposure. Treatments;—Dilution Water Control (DWC), Solvent Control (SC) and 62.5, 125, 250 and 500 ng/L of either MT or DHT.

*B*. *glabrata* were exposed to either MT or DHT during development. At the end of the 30 days exposure, snail survival varied between treatments, doses and, in some cases, between replicate tanks (Fig 2A and 2B). Overall survival in the DHT experiment was 61.7% and the numbers of snails surviving in each replicate tank were similar to one another (Fig 2B and S2 File; Table A). In the MT experiment overall survival was high (80.4%). However, more variation between replicate tanks was observed; with more than double the number of snails surviving in one 250 ng/L MT replicate tank compared to the other (Fig 2A and S1 File; Table A). In both experiments survival generally decreased with increasing dose (Fig 2A and 2B), however this was more marked in the DHT study where maximum survival was 97.5% (39 out of 40) in the DHT-SC and fell to 5% (i.e. one individual in each replicate tank) in the 250 ng/L DHT treatment.

Snails from the whole MT experiment had shell diameters ranging from 2.97 to 15.03 mm. In the DWC tanks snails shell diameter ranged from 7.62 to13.43 mm, whereas in all the other treatments shell size varied considerably more (S1 File; Table B); Individual weight of snails from the whole MT experiment ranged from 0.02 to 0.69 g and, as might be expected, weight mirrored shell diameter (S1 File; Table B) with snails from the DWC having a smaller range than other treatments in this experiment. There were significant differences in shell diameter and/or total weight between replicate tanks in four-out-of-six treatments (DWC, SC, 125 ng/L MT and 250 ng/L MT. S1 File; Table A), which precluded further statistical analysis grouped by treatment. Analysis by tank (Tukey test) showed no clear association of MT dose and snail size (S1 File; Tables C and D).

Shell diameters in the DHT exposure ranged from 6.78 to 15.76 mm over the whole experiment, wet weight ranged from 0.11 g to 0.70 g, in this experiment individual treatments generally showed similar variation in size and weight (S1 File; Table B). There was a significant difference between replicate tanks in shell diameter and total weight in the 62.5 ng/l DHT treatment (S2 File; Table A), which precluded further statistical analysis grouped by treatment. Analysis by tank (Tukey test) showed no clear association of DHT dose and snail size (S2 File; Tables C and D).

To assess possible impacts of androgens (MT or DHT) on somatic development, linear regression of shell diameter and total body weight (square root transformation of weight) for each treatment was conducted. Significant linear correlations were observed overall for both the MT (R^2^ = 0.9445, P = 0.001) and DHT (R^2^ = 0.8922 P = 0.003) studies, as expected. No obvious deviation from the expected pattern of growth was found for any of the treatments, i.e. all treatments followed the same linear growth pattern as the controls (S3A and S3B Fig).

#### Quantitative assessment of the size of reproductive glands

**Fig 3:**
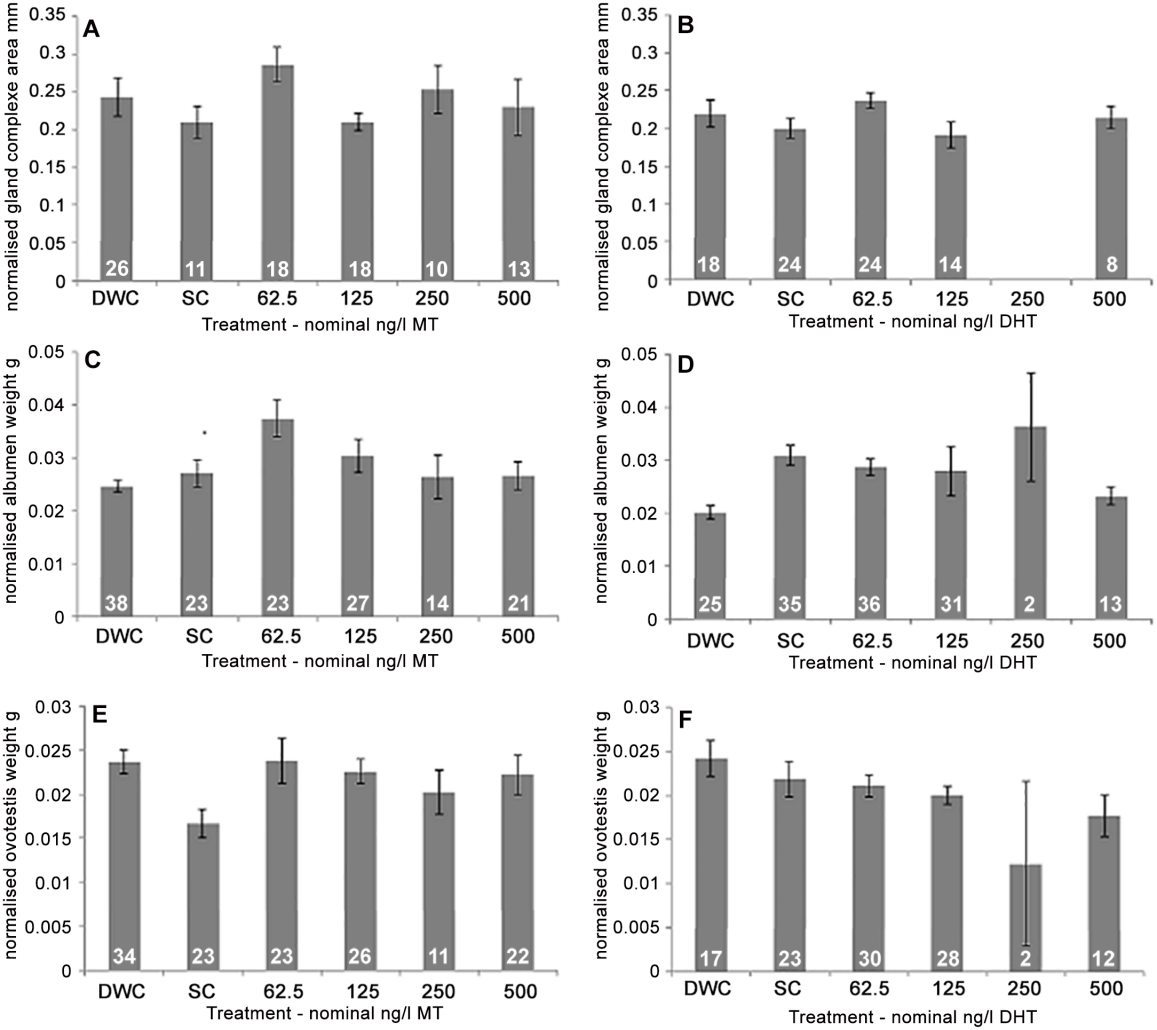
Average normalized size of reproductive tissues from *B*. *glabrata* developmentally exposed to methyltestosterone or dihydrotestosterone. Normalized surface area (mm) of glandular complex (A, B), normalized albumen gland weight (g) (C, D) and normalized ovotestis weight (g) (E, F) from *B*. *glabrata* developmentally exposed to MT (A, C, E) or DHT (B, D, F) through to sexual maturity (30-day exposure). Error bars represent standard error. White numbers within bar indicate sample size (n). Treatments;—Dilution Water Control (DWC), Solvent Control (SC) and 62.5, 125, 250 and 500 ng/L of either MT (A, C, E) or DHT (B, D, F).

To assess possible androgenic effects on growth and development of reproductive glands in developmentally exposed *B*. *glabrata*, the size (mm^2^) or weight (g) of reproductive tissues were compared. In both the MT and DHT studies there were no significant differences between replicate tanks for normalized gland complex size. No significant differences were found between treatments in either the MT or DHT exposures (P = 0.297 and 0.362, respectively) and, overall, no dose dependent trend was observed for the glandular complex for either study (Fig 3A and 3B). No comparison could be made for the 250 ng/L DHT exposure due to low survival (n = 2).

In the MT study, a significant difference, between replicate tanks, in the size of the albumen gland was observed in both the 62.5 ng/L and 125 ng/L MT treatments (P = 0.034 and 0.042). Consequently, further statistical analysis of treatment could not be conducted. Analysis by tank (Tukey test) showed no clear association of MT exposure and albumen gland size (S1 File; Table J) and no trend in albumen size was observed (Fig 3C). In the DHT exposure study no significant differences were found for normalized albumen gland weight between replicate tanks, and no significant differences (P = 0.072) were found between treatments (Fig 3D); again no comparison could be made for the 250 ng/L DHT exposure.

In the MT study, a significant difference was found between replicates in the solvent control (SC) treatment (P = 0.023) for normalized ovotestis weight, consequently, further statistical analysis of treatment could not be conducted. However, analysis by tank (Tukey test) showed no clear association of MT exposure and ovotestis weight (S1 File; Table G) and no trend relating to MT exposure was observed (Fig 3E). In the DHT-exposed snails, no significant differences between replicates were found for normalized ovotestis weight, and overall, no significant (P = 0.111) effect of exposure was found on ovotestis weight (Fig 3F). As before, no comparison could be made for the 250 ng/L DHT exposure.

#### Histopathological analyses of reproductive tissues

To assess possible androgenic effects on reproductive tissue structure or cell type, histopathology was conducted on reproductive tissues (albumen gland, glandular complex and ovotestis) from developmentally exposed *B*. *glabrata* from both MT and DHT studies. Histopathology was conducted on the ovotestis, albumen gland and glandular complex of exposed and control snails (>5 mm in shell diameter) from both the MT and DHT exposures.

The histopathological analyses from the MT (S4A–S4F Fig) and DHT (S5A–S5E Fig) studies reveal no compound/dose related morphological changes in the albumen gland. In some individuals the glandular epithelial cells were heterogeneously stained, but this was observed in all treatments (including DWC) irrespective of the dose. This could be due to variation in the granules and cytoplasm present in the secretory cells, depending on the egg laying process [38].

The GC comprised of membrane gland, mucous gland and prostate gland (S6A–S6F and S7A–S7E Figs). The histological sections of GC from MT study and DHT study showed no chemical-related or dose-related morphological changes.

Ovotestis from the MT and DHT exposures were initially assessed for dose related effect on oogenesis and spermatogenesis. There were no treatment related effects (S8 and S9 Figs); all stages of oogenesis and spermatogenesis, as described by De Jong-Brink *et al*. [39,40], were present.

### Biological effects of exposure: gene expression analyses

#### Screening for differentially expressed genes

From the morphological and histopathological analysis (above) no gross effects on reproductive glands or tissues were found when *B*. *glabrata* were exposed to either MT or DHT. However, a number of other publications have shown MT to impact gastropod mollusk reproduction and gametogenesis [14,16,41,42] and therefore molecular analysis were completed using tissues from the MT exposed snails.

**Fig 4:**
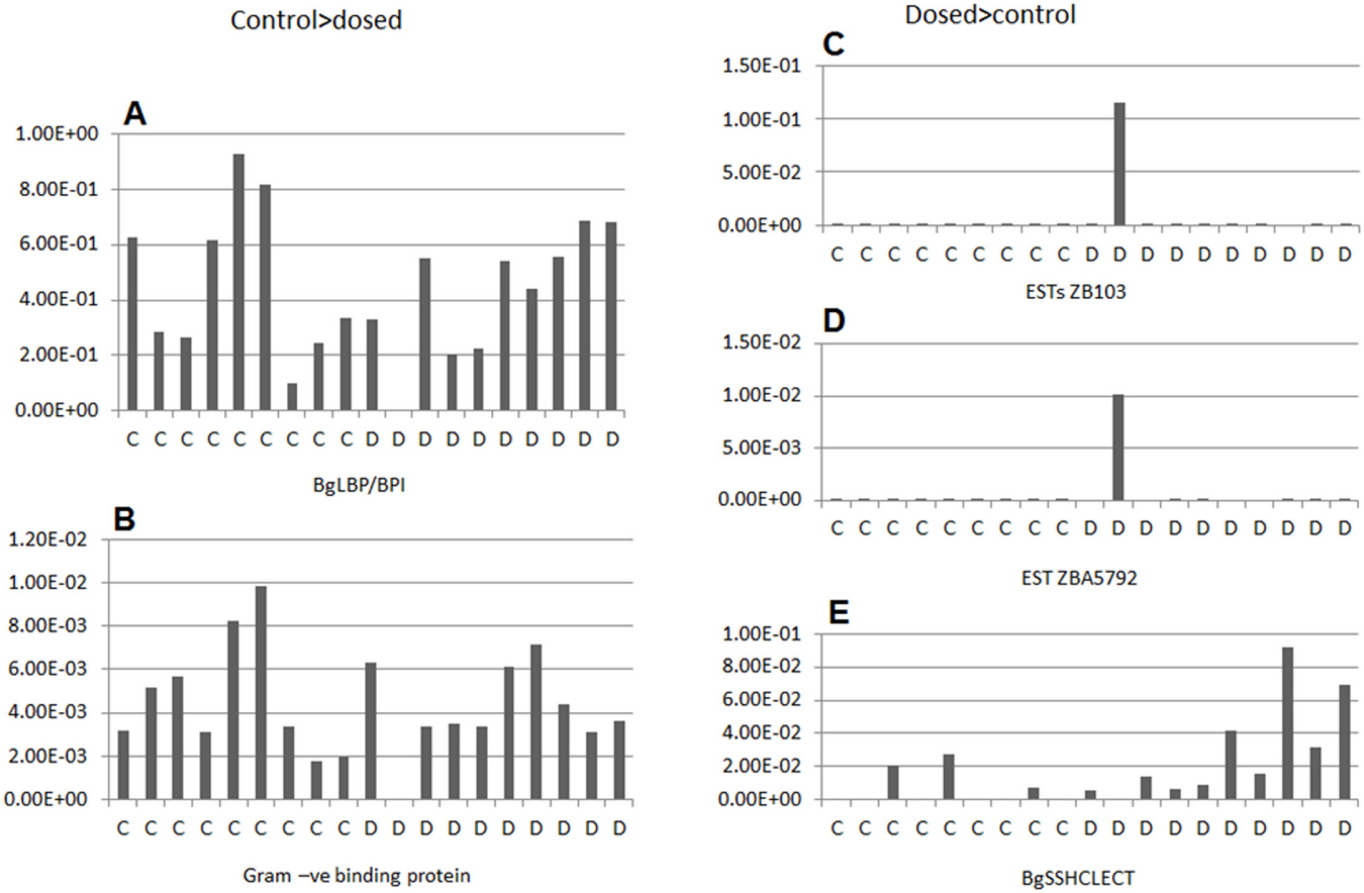
Normalized gene expression of individual albumen glands from 125ng/l-MT and solvent-control groups. Normalized gene expression in individual snails for 5 genes identified as differentially expressed in the albumen glands of exposed and control groups. Ten individual snails were included in the analysis from each treatment (10 MT-dosed; D and 10 solvent-control; C). qPCRs were performed in triplicate and normalized to 18S.

To identify differentially expressed transcripts for use as biomarkers for androgen exposure, or to help elucidate mechanisms for androgen effects, suppression subtractive hybridization (SSH) was used to compare reproductive tissues taken from solvent control and MT-exposed *B*. *glabrata*. Four subtracted clone libraries, enriched for MT exposed- or MT solvent control-specific transcripts, were made for the albumen and ovotestis, using 10 individual snails exposed to 125 ng/L MT compared to 10 from the solvent control. The dose (125 ng/L) was selected as the highest dose before higher mortality rates were observed, and the aim was to identify possible molecular markers of androgen (endocrine) modulation, and not toxicity markers. Differential screening identified 24 dosed-specific transcripts and 25 control-specific transcripts from the albumen libraries (i.e. transcripts whose expression was reduced in the dosed tissues) and 6 dosed-specific and 3 control-specific transcripts, from the ovotestis libraries. These were sequenced and the resulting sequences screened for duplicates and SSH sequence artifacts (poly SMART primer). Three unique sequences were identified from the MT dosed albumen gland and 2 from the MT control albumen gland (Table 2). None were identified from ovotestis (all the sequences were a poly SMART primer artifact). Homology searches (Table 2) indicated that, of the 3 MT dosed specific transcripts from the albumen gland, 2 had no known protein homologues, although they matched (Expect value E = 0) previously sequenced *B*. *glabrata* ESTs (GenBank: DY523257.1 (and 29 other ESTs) and CV548292.1). The other transcript identified (E = 0) a c-type lectin previously identified in *B*. *glabrata* (EB709537.1). The control-specific transcripts were identical to a previously sequenced lipopolysaccharide binding protein/bactericidal permeability increasing protein (GenBank: KC206037; [43]) and a gram-negative binding protein (GenBank:EF452345.1; [44]). Quantitative PCR using specific primers was carried out to determine if gene expression levels for the differentially expressed transcripts, found in the pooled samples, were differentially expressed across all individuals. However, qPCR indicated that one specific individual, exposed to MT, was biasing the libraries (Fig 4) even though equal amounts of RNA had been pooled. Therefore, although the genes identified might represent a response to MT, this was not consistent across all the samples and none would therefore make a useful biomarker or indicator of androgen exposure.

**Table 2:** Identification of sequenced SSH fragments from albumen gland in the MT exposure experiment.

Fragment	Library	Size (bp)	Best BlastN match (NCBI NR/ESTs)	Species	Acc. No	dbEST Acc No
MTA34	control	664	BgLBP/BPI1	*B*. *glabrata*	KC206037.1	JZ875077
MTA41	control	481	Gram negative binding protein	*B*. *glabrata*	EF452345.1	JZ875078
MTA21	dosed	743	Unknown EST	*B*. *glabrata*	EW997484.1	JZ875075
MTA18	dosed	687	Unknown EST	*B*. *glabrata*	CV548292.1	JZ875074
MTA23	dosed	527	BgSSH CLECT	*B*. *glabrata*	EB709537.1	JZ875076

## Discussion

Our hypothesis was that *B*. *glabrata* might show a consistent physiological or genetic response to androgens, making them suitable as a model test organism. When taken together, our results provide ample evidence to refute this hypothesis. The primary aim of the *in vivo* developmental exposure to two potent androgens, 5α-dihydrotestosterone (DHT) and 17α-methyltestosterone (MT); was to investigate whether developmental exposure to these androgens would affect the growth of reproductive glands and organs in *B*. *glabrata* and/or whether specific molecular markers of androgen exposure could be determined in a mollusk, thus providing an invertebrate alternative model for male reproductive toxicity testing. However we found no consistent effects on *B*. *glabrata*.

### Effect of androgens on survival and growth

Hatchling survival in both the DHT and MT studies was lower in the 250 ng/L and 500 ng/L (MT or DHT) than the dilution water control (DWC) or solvent control (SC) treatments. This suggests there may have been some toxicity to the developing snails at these concentrations (although mortality in the MT study was generally lower than in the DHT study). Toxicity has not been previously reported in other gastropod species exposed to similar nominal concentrations of androgens [14,16,41,42,45], and the dosing series for these experiments were designed to mimic concentrations documented to have endocrine effects in mollusks [14,16,41] and aquatic vertebrates [32,33] i.e. in the tens to hundreds of ng/L range. However, the experimental design of earlier work with mollusks differs from this study, in that many were static-renewal rather than continuous dosing. Investigators measuring exposure concentrations in static renewal systems often find much lower (sometimes <20%) than nominal exposure concentrations [17,45], whereas in this study exposure concentrations of 80–110% nominal MT and 65–70% nominal DHT where measured. Therefore snails in our experiments may have been exposed to higher concentrations than similar species in other studies.

In vertebrates, exposure to androgens has been reported to increase somatic growth; for example in developmentally exposed fathead minnows (*Pimephales promelas*), DHT significantly increased weight and length in males (20 ng/L DHT) and females (200 ng/L DHT) [33]. No dose dependent trend could be identified for shell size or total weight in the DHT study; on average the smallest snails were found in the 62.5 ng/L DHT treatment, and the largest in the 125 ng/L DHT treatment (S2 File; Table B). In the MT study, the largest snails were found in the DWC treatment and the smallest in the 250 ng/L MT (S1 File; Table B), and as with the DHT study, no dose dependent trend could be seen.

*B*. *glabrata*, like other pulmonate gastropods, is a typical r-selection reproductive strategist, with large numbers of offspring and relatively high levels of mortality. Therefore, it was expected that a proportion of the hatchlings (~30% from unpublished preliminary work in our laboratory) would not survive to reproductive age, thus reducing the density in the exposure tanks over time. As mortality levels were variable, there were differences in densities of the snails between some of the tanks, perhaps contributing to variable growth rates between tanks. Even in replicate DWC tanks, however, with similar survival and densities, significant differences were found in shell size and total weight of the snails i.e. DWC tanks from the MT study (S1 File; Table A). To account for these variations in total weight of individual snails between tanks, measurements of size and/or weight of the glandular and ovotestis tissues were normalized by individual total weight prior to statistical analysis.

### No consistent dose-related effects of androgens on reproductive tissue size and histopathology

One of the primary markers of androgen exposure in vertebrate models, such as the Hershberger test, is increased size/weight of male specific organs. For example, in a pubertal rat assay (enhanced OECD test guideline 407) MT exposure resulted in increased prostate weight in males treated with 200 mg/kg/day [46]. In contrast, no significant effect on normalized Glandular Complex (GC) (including prostate gland) size was found for *B*. *glabrata* snails exposed to either MT or DHT. There were also no consistent significant dose-related effects on normalised albumen gland weight in the MT or DHT exposure studies. Although the albumen glands of the 62.5 ng/L and 125 ng/L MT exposed snails were much larger than those from the DWC, SC, 250 ng/L and 500 ng/L treated snails, there were significant intra-replicate differences in albumen gland weight in both the 62.5 and 125 ng/L treatments (S1 File; Table H), suggesting this was not an effect of MT exposure, but an artefact or ‘tank’ effect. In the DHT study, there were no such within-replicate differences and no significant effects of androgen exposure on albumen gland weight. In general, gastropod albumen gland size is thought to be related to accumulation of secretory products in the tissue, due to different stages of the egg-laying cycle [47,48]. Enlarged albumen glands were reported just before egg laying in *Helisoma duryi* [49] and in *L*. *stagnalis* egg laying status has also been shown to affect albumen gland size with lower weights in those snails that had laid more eggs [50]. This factor could account for the variation in albumen gland size in the MT study, as some individuals may have laid eggs whereas others had not.

In vertebrate models, histopathological changes to gland structure or development are also reported in androgen-exposed individuals. For example, in pubertal male rats, histology of the seminal vesicle and prostate gland show enhanced glandular development (after 1 mg/kg/day Testosterone Propionate by subcutaneous injection) [51]. In comparison, no histological impact of MT or DHT exposure was observed in the albumen gland or the Glandular Complex (GC) of androgen-exposed *B*. *glabrata*. Differences were observed between individual snails in the staining of the epithelium of the tubules in the albumen gland, but these variations were found across all treatments, and were due to the presence of more vacuolated cytoplasm and less secretory granules in the epithelium [47,52], possibly due to variation in the egg-laying cycle of the snails at the time of sampling. A varying appearance in the structure, such as dilation of the tubules of the albumen gland, has been linked with different physiological states due to metabolic processes and sexual activity in *L*. *stagnalis* [53] and has been reported in control snails by Czech *et al*. [16]. The lack of effects of MT or DHT exposure on secondary sexual glands in *B*. *glabrata* is somewhat in contrast to previously reported experiments with *L*. *stagnalis*, which showed a low level of degeneration to the albumen and prostate glands after 8 weeks exposure to 100 ng/L MT in adult snails [16]. Differences in species and the timing of exposure (adult vs. juvenile) may account for the different observations between our study and that of Czech *et al*. [16].

In a range of vertebrate models, androgen exposure has been documented to impact gonadal development. For example, female pubertal rats exposed to 200 mg/kg/day MT had significantly reduced ovary weight [46]. Japanese Medaka (*Oryzias latipes*), developmentally exposed to androgens via the water, in a similar way to *B*. *glabrata* in this study, exhibited male skewed sex ratios and testis-ova at average concentrations of 27.75 ng/L MT [54]. In another study juvenile *P*. *promelas* exposed to 20 and 200 ng/L of DHT for 45 days induced significant advancement in spermatogenesis in males and caused sexual disruption in females [33].

No differences in oogenesis or spermatogenesis were observed in any of the MT or DHT exposed snails compared to controls and no significant effect of androgen exposure (MT or DHT) was found on the ovotestis weight. The lack of gross histopathological changes to the ovotestis in *B*. *glabrata* exposed to MT is in contrast to the recent work of Rivero-Wendt *et al*. [42], in which adult *B*. *glabrata* were exposed semi-statically to MT (0.01 mg/L to 1 mg/L MT). Rivero-Wendt *et al*. [42] reported that nominal MT concentrations of 0.1 mg/L and 1.0 mg/L significantly increased the area of ovotestis acini occupied by mature spermatozoa, compared to the water control. This might suggest much higher concentrations of MT than used in our study (mg/L vs. ng/L) could stimulate spermatogenesis. However, it is also worth noting that in their study the solvent control (0.001% ethanol) snails showed the largest average area occupied by sperm (almost double that of the water control). This suggests MT may not have been the overriding factor in increasing sperm production in these snails and highlights the importance of considering possible solvent mediated affects in toxicology studies [55].

### Gene expression analyses in MT-exposed snails

In previous studies in fish, SSH has been used to identify possible androgen responsive genes *in vitro* [56] and *in vivo* [57]. We identified differences in gene expression using SSH between pooled *B*. *glabrata* albumen gland from MT-exposed (125 ng/L MT) and solvent control samples. However, subsequent investigation of expression of each gene in individual snails revealed that these differences were mainly attributable to one individual, even though equal amounts of RNA had been pooled. This one individual may have been responding to MT, however the difference could be due to other factors, such as whether the individual had just laid an egg mass. The lack of a consistent effect makes the identified genes unsuitable for use as markers of androgen exposure. No differences in gene expression were found between control and MT-exposed ovotestis.

### Significance of the negative finding

The absence of significant dose-dependent physiological (DHT and MT) or molecular responses (MT) on *B*. *glabrata* was surprising considering the number of publications that suggest gastropod mollusks have physiological levels of vertebrate-type androgens naturally occurring in their tissues, use vertebrate-type androgens in reproduction or are reproductively disrupted by exogenous androgen or anti-androgen exposure [11,14,15,25,27,42,58]. For example, in the freshwater prosobranch gastropod, *Marisa cornuarietis*, MT showed masculinizing (imposex) effects in adult female snails from 100 ng/L (nominal) to complete virilization at 1 μg/L (nominal) [11], and disruption to both spermatogenesis, oogenesis and overall fecundity above nominal concentrations of 100 ng/L MT [11]. However, the majority of publications documenting effects of androgens are in prosobranch (generally gonochoristic) gastropods rather than pulmonate (hermaphrodite) gastropods such as *B*. *glabrata*. Considering the large evolutionary divergence of gastropods it might not be surprising that they show different responses. However, a number of studies have suggested either a role of vertebrate androgens in pulmonate gastropod reproduction in *Biomphalaria alexandrina* [25] or stimulatory effects of androgens to reproductive tissues in *B*. *glabrata* [42]. As previously discussed, Rivero-Wendt and colleagues may have identified a solvent effect on spermatogenesis, rather than an effect of steroid androgens. Evidence for the localization of the androgen receptor in *B*. *alexandrina* was obtained using a monoclonal antibody for the mouse protein [25], which, since we now know that there is no snail AR homologue [29], may have been due to non-specific or incorrect binding in the snail.

Recent reviews on vertebrate sex steroids in mollusks by Scott [26,59] also question the validity and robustness of published studies on this topic. For instance, Scott [59] documents the following: of the 17 studies which report reproductive or endocrine effects of steroid androgens in mollusks none had within study replication (e.g. replicate dosed tanks) and only one study measured the concentration of chemical dosed into the tank to verify exposure level. Indeed, by using replication and assessing within-treatment variation in our study we found variation between replicate tanks could be more significant than those between treatment concentrations, allowing us to discount possible chemical mediated effects as the driver of the variation. This may not have been apparent if we had only used one tank per test concentration.

In a recent publication by Giusti *et al*.[60] (in another pulmonate gastropod *L*. *stagnalis*), which measured exposure concentrations and used adequate replication, it was found that although testosterone altered the expression of 49 proteins in *L*. *stagnalis*, many of these proteins were considered to be modulated as a general toxicity response. Specific reproductive proteins such as Piwi; a germline stem-cell protein, and ovipostatin; a protein produced in the prostate, were not found to be significantly altered by exposure to testosterone, although they were altered by other known vertebrate endocrine disruptive compounds [60] via, as yet, unidentified mechanisms. Indeed, neither Giusti *et al*. [17,45] nor Czech *et al*. [16] found significant reproductive effects of testosterone or methyltestosterone in adult *L*. *stagnalis*, suggesting vertebrate type androgens do not regulate reproduction in this species. Consequently Giusti *et al*. [45] concluded that testosterone would not be a good positive control in reproductive chemical testing in *L*. *stagnalis*. These reports in adult pulmonate gastropods, as well as the recent finding that *B*. *glabrata* draft genome [29] contains no convincing orthologues for the AR, support our finding of no significant effect of steroid androgens on *B glabrata* reproductive physiology.

## Conclusion

In summary, we assessed gross morphology, histopathology and gene expression to identify possible effects of androgens on developing *B*. *glabrata*. We confirmed that the snails were exposed to the compounds in the expected nominal range and used within study replication. However, none of these techniques showed significant effects of androgen exposure, demonstrating that growth and development of the reproductive tract in *B*. *glabrata* is unaffected by developmental exposure (embryo through to sexual maturity) to either MT or DHT, both of which are potent androgens in vertebrates. The paucity of physiological and molecular effects of androgen exposure in *B*. *glabrata* in our laboratory is supported by systematic genomic searches for the androgen receptor in the preliminary *B*. *glabrata* genome, where we found no convincing orthologues for the androgen receptor despite identifying 39 other nuclear receptors representing all major NR groups [29]. The consequence of these findings are significant and suggest *B*. *glabrata* and possibly other mollusks species would not be appropriate models for testing androgenic or anti-androgenic compound and that this species could not be used as a replacement for vertebrates in testing androgenic or anti-androgenic compounds toxicity or mode of action testing research. Although outside of the scope of the *B*. *glabrata* research presented here, investigation of the functions of other mollusk nuclear receptors may produce further understanding of general mollusk endocrinology and underpin mechanistically appropriate methods of using mollusks to support the 3Rs strategy in reducing and replacing vertebrates in biomedical research.

## Supporting Information

S1 Fig Annotated photograph of reproductive tract in *B*. *glabrata*. At the distal end of the final whorl in *B*. *glabrata*, is the ovotestis that leads into the hermaphrodite duct then to the carrefour where fertilization occurs. Albumen gland (red circle) is the female accessory reproductive gland that pours its secretions into the carrefour, providing nutrition to growing embryo. The glandular complex (GC) comprises of both male (prostate and sperm duct) and female accessory glands (mucous gland and oothecal gland).
(TIF)

S2 Fig Photomicrographs of *B*. *glabrata* reproductive tissues from stock snails (non-exposed) for comparison of fixation methods. Albumen gland (A-B), Glandular complex (C-D), and Ovotestis (E-F) preserved in either Bouin’s fixative (A, C, E) or in RNAlater followed by Bouin’s fixative (B, D, F). RNAlater fixation in both glandular tissues and ovotestis resulted in loss/disruption of connective tissues, basement membranes and fat cells. Ct: Connective tissue; Ept: Epethelium tissue; Ooc: Oocyte; Lumen: Lumen of a gland tubule; Sc: Sertoli cell; Spg: Spermatogonia; Spd: Spermatid; Spz: Spermatozoa.
(TIF)

S3 Fig Regression of shell diameter against total body weight from A) MT or B) DHT exposures. Significant positive correlation between snail size (shell diameter, mm) and weight (g, Square root transformed) was seen in both treatments. Each snail is represented on the graph; Dilution Water control (DWC)—blue diamonds, Solvent Control (SC)—red diamonds, 62.5 ng/L MT or DHT—green triangles, 125 ng/L MT or DHT—purple crosses, 250 ng/L MT or DHT—blue double crosses, and 500 ng/L MT or DHT—orange dots. Linear trend line, Regression coefficient (R^2^) and significance (P) are highlighted on each graph.
(TIF)

S4 Fig Photomicrographs of representative albumen gland (AG) from *B*. *glabrata* developmental exposed to Methyltestosterone (MT). Representative photomicrographs; A) from dilution water control (DWC), B) Solvent control (SC), C) 62.5 ng/L MT, D) 125 ng/L MT, E) 250 ng/L MT and F) 500 ng/L MT. Scale bar 100 μm in each case. The AG in *B*. *glabrata* consists of densely packed tubules consisting of columnar glandular epithelial cells that were heterogeneously stained in some individuals from all treatments (including DWC) irrespective of the dose. Therefore no treatment related effects to the albumen gland of the MT exposed groups were observed as compared to controls (either DWC or SC). Ct: Connective tissue; Ept: Epethelium tissue; Lumen: Lumen of a gland tubule.
(TIF)

S5 Fig Photomicrographs of representative albumen gland (AG) from *B*. *glabrata* developmental exposed to Dihydrotestosterone (DHT). Representative photomicrographs; A) from dilution water control (DWC), B) Solvent control (SC), C) 62.5 ng/L DHT, D) 125 ng/L DHT and F) 500 ng/L DHT. (250ng/L treatment excluded due to high mortality) Scale bar 100 μm in each case. As before in MT treatment, the glandular epithelial cells were heterogeneously stained in some individuals, but this was observed in all treatments (including DWC) irrespective of the dose. Therefore no treatment related effects to the albumen gland of the DHT exposed groups were observed as compared to controls (either DWC or SC). Ct: Connective tissue; Ept: Epethelium tissue; Lumen: Lumen of a gland tubule.
(TIF)

S6 Fig Photomicrographs of representative glandular complex (GC) from *B*. *glabrata* developmental exposed to Methyltestosterone (MT). Representative photomicrographs; A) from dilution water control (DWC), B) solvent control (SC), C) 62.5 ng/L MT, D) 125 ng/L MT, E) 250 ng/L MT and F) 500 ng/L MT. Scale bar 100 μm in each case. GC comprises of mucous gland, membrane gland and prostate gland. Due to RNAlater fixation, tissues stained a darker colour in all the experimental samples and a slight disruption of connective tissue and basement membrane was observed. No significant treatment related effects to any of the three glands in the glandular complex were detected in MT exposed snails compared to the control (either DWC or SC).
(TIF)

S7 Fig Photomicrographs of representative glandular complex (GC) from *B*. *glabrata* developmental exposed to Dihydrotestosterone (DHT). Representative photomicrographs; A) from dilution water control (DWC), B) solvent control (SC), C) 62.5 ng/L MT, D) 125 ng/L MT, and E) 500 ng/L MT (250ng/L treatment excluded due to high mortality). Scale bar 100 μm in each case. As seen in MT treatment, no significant treatment related effects to any of the three glands in the glandular complex were observed in DHT exposed snails compared to the control (either DWC or SC).
(TIF)

S8 Fig Photomicrographs of representative of ovotestis (OT) from *B*. *glabrata* developmental exposed to Methyltestosterone (MT). Representative photomicrographs; A) from dilution water control (DWC), B) solvent control (SC), C) 62.5 ng/L MT, D) 125 ng/L MT, E) 250 ng/L MT and F) 500 ng/L MT. Oo: Oocyte; Ser: Sertoli cell; Spg: Spermatogonia; Spd: Spermatid; Spz: Spermatozoa.
(TIF)

S9 Fig Photomicrographs of representative of ovotestis (OT) from *B*. *glabrata* developmental exposed to Dihydrotestosterone (DHT). Representative photomicrographs; A) from dilution water control (DWC), B) solvent control (SC), C) 62.5 ng/L MT, D) 125 ng/L MT, and E) 500 ng/L MT. (250ng/L treatment excluded due to high mortality) Oo: Oocyte; Ser: Sertoli cell; Spg: Spermatogonia; Spd: Spermatid; Spz: Spermatozoa.
(TIF)

S1 File Tables of descriptive data and statistical analysis for *B*. *glabrata* from the Methyltestosterone study. (PDF)

S2 File Tables of descriptive data and statistical analysis for *B*. *glabrata* from the Dihydrotestosterone study. (PDF)
