# Supplementary figures and images for: Steroid Androgen Exposure during Development Has No Effect on Reproductive Physiology of Biomphalaria glabrata
Source: PLoS One. 2016 Jul 22;11(7):e0159852. doi: 10.1371/journal.pone.0159852 (PMC4957768; doi:10.1371/journal.pone.0159852)

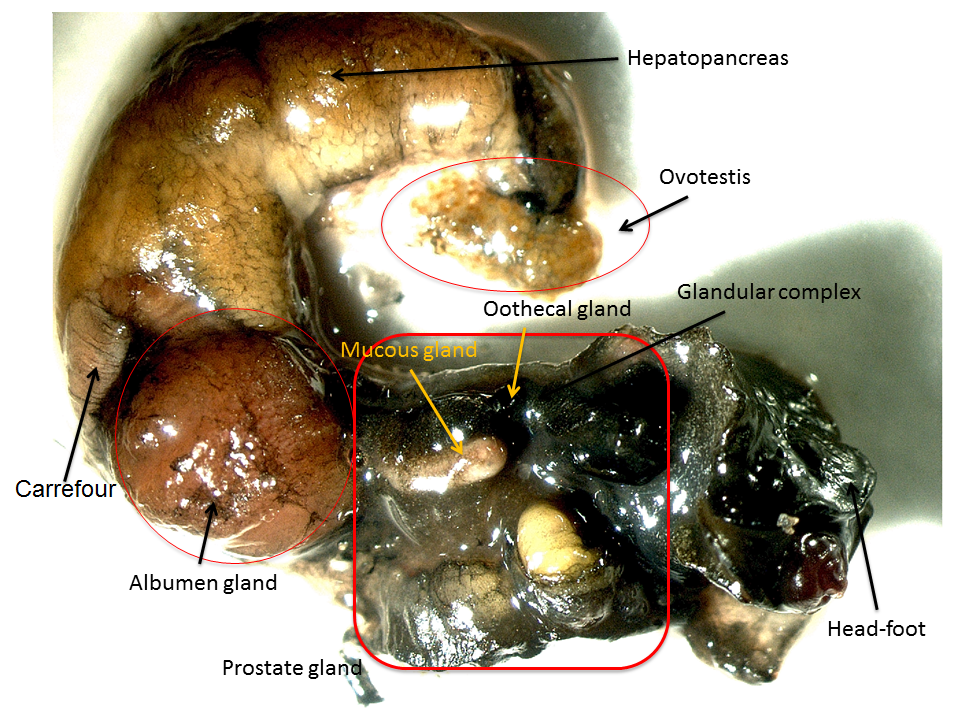

Supplement: S1 Fig — At the distal end of the final whorl in B. glabrata, is the ovotestis that leads into the hermaphrodite duct then to the carrefour where fertilization occurs. Albumen gland (red circle) is the female accessory reproductive gland that pours its secretions into the carrefour, providing nutrition to growing embryo. The glandular complex (GC) comprises of both male (prostate and sperm duct) and female accessory glands (mucous gland and oothecal gland). (TIF) [file pone.0159852.s001.tif]

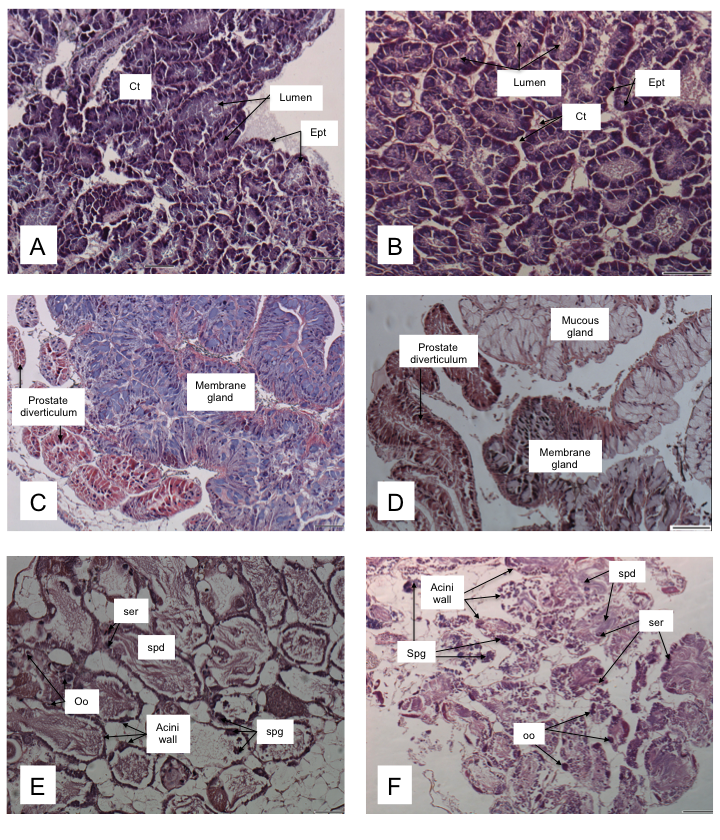

Supplement: S2 Fig — Albumen gland (A-B), Glandular complex (C-D), and Ovotestis (E-F) preserved in either Bouin’s fixative (A, C, E) or in RNAlater followed by Bouin’s fixative (B, D, F). RNAlater fixation in both glandular tissues and ovotestis resulted in loss/disruption of connective tissues, basement membranes and fat cells. Ct: Connective tissue; Ept: Epethelium tissue; Ooc: Oocyte; Lumen: Lumen of a gland tubule; Sc: Sertoli cell; Spg: Spermatogonia; Spd: Spermatid; Spz: Spermatozoa. (TIF) [file pone.0159852.s002.tif]

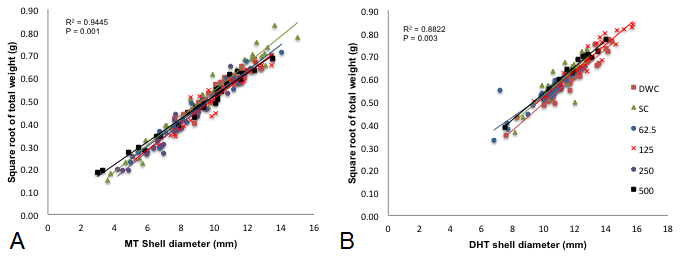

Supplement: S3 Fig — Significant positive correlation between snail size (shell diameter, mm) and weight (g, Square root transformed) was seen in both treatments. Each snail is represented on the graph; Dilution Water control (DWC)—blue diamonds, Solvent Control (SC)—red diamonds, 62.5 ng/L MT or DHT—green triangles, 125 ng/L MT or DHT—purple crosses, 250 ng/L MT or DHT—blue double crosses, and 500 ng/L MT or DHT—orange dots. Linear trend line, Regression coefficient (R2) and significance (P) are highlighted on each graph. (TIF) [file pone.0159852.s003.tif]

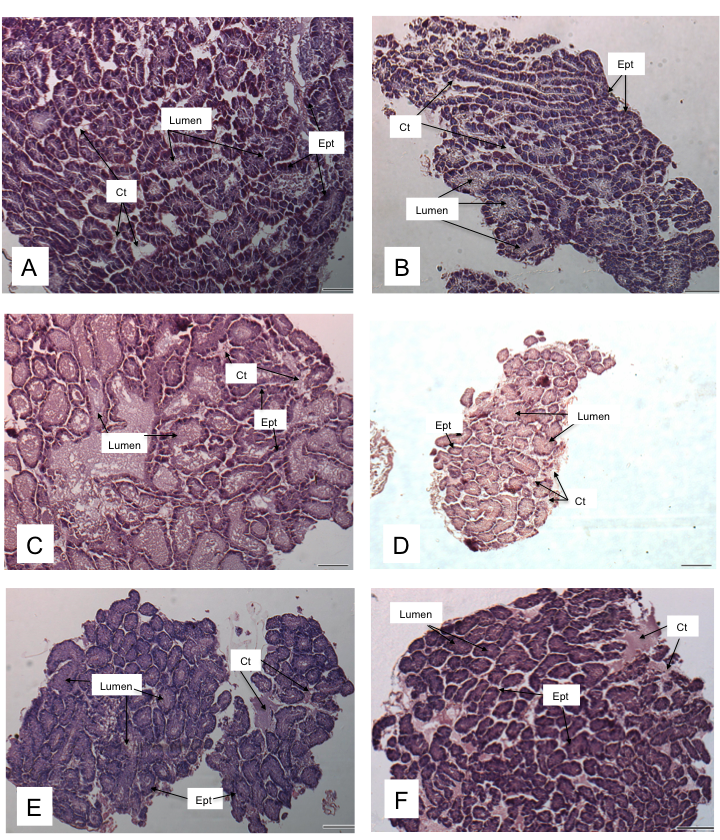

Supplement: S4 Fig — Representative photomicrographs; A) from dilution water control (DWC), B) Solvent control (SC), C) 62.5 ng/L MT, D) 125 ng/L MT, E) 250 ng/L MT and F) 500 ng/L MT. Scale bar 100 μm in each case. The AG in B. glabrata consists of densely packed tubules consisting of columnar glandular epithelial cells that were heterogeneously stained in some individuals from all treatments (including DWC) irrespective of the dose. Therefore no treatment related effects to the albumen gland of the MT exposed groups were observed as compared to controls (either DWC or SC). Ct: Connective tissue; Ept: Epethelium tissue; Lumen: Lumen of a gland tubule. (TIF) [file pone.0159852.s004.tif]

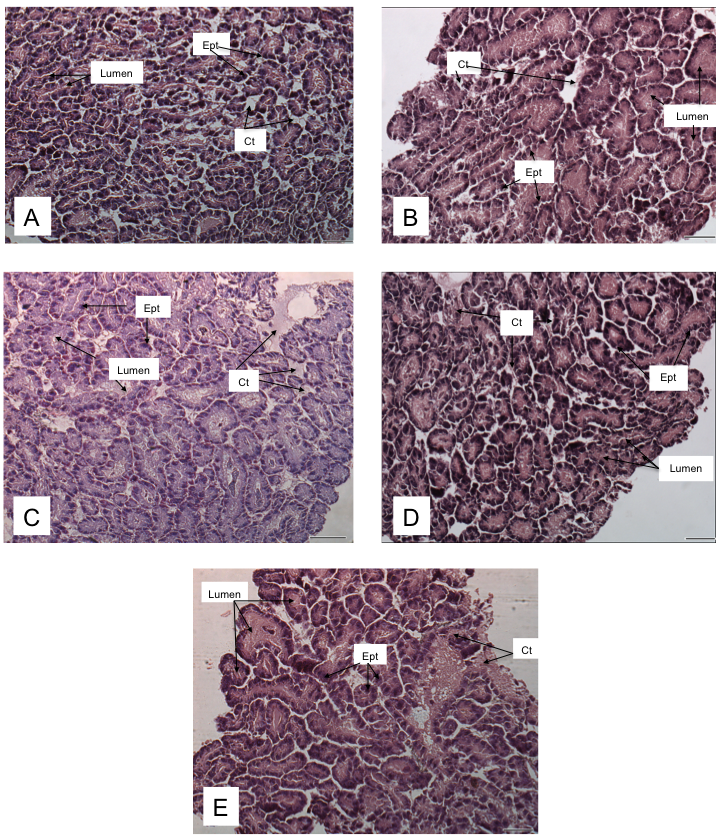

Supplement: S5 Fig — Representative photomicrographs; A) from dilution water control (DWC), B) Solvent control (SC), C) 62.5 ng/L DHT, D) 125 ng/L DHT and F) 500 ng/L DHT. (250ng/L treatment excluded due to high mortality) Scale bar 100 μm in each case. As before in MT treatment, the glandular epithelial cells were heterogeneously stained in some individuals, but this was observed in all treatments (including DWC) irrespective of the dose. Therefore no treatment related effects to the albumen gland of the DHT exposed groups were observed as compared to controls (either DWC or SC). Ct: Connective tissue; Ept: Epethelium tissue; Lumen: Lumen of a gland tubule. (TIF) [file pone.0159852.s005.tif]

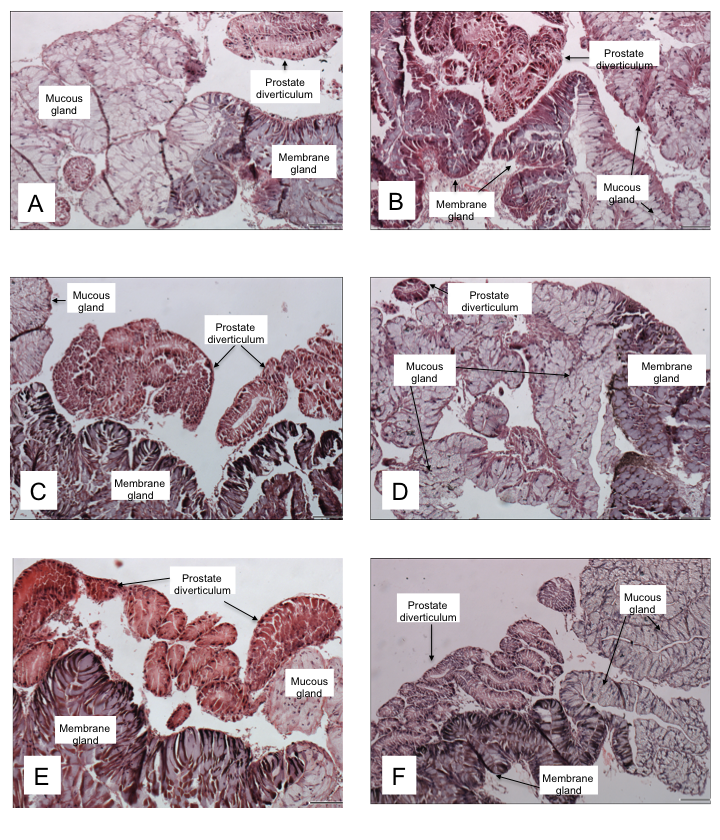

Supplement: S6 Fig — Representative photomicrographs; A) from dilution water control (DWC), B) solvent control (SC), C) 62.5 ng/L MT, D) 125 ng/L MT, E) 250 ng/L MT and F) 500 ng/L MT. Scale bar 100 μm in each case. GC comprises of mucous gland, membrane gland and prostate gland. Due to RNAlater fixation, tissues stained a darker colour in all the experimental samples and a slight disruption of connective tissue and basement membrane was observed. No significant treatment related effects to any of the three glands in the glandular complex were detected in MT exposed snails compared to the control (either DWC or SC). (TIF) [file pone.0159852.s006.tif]

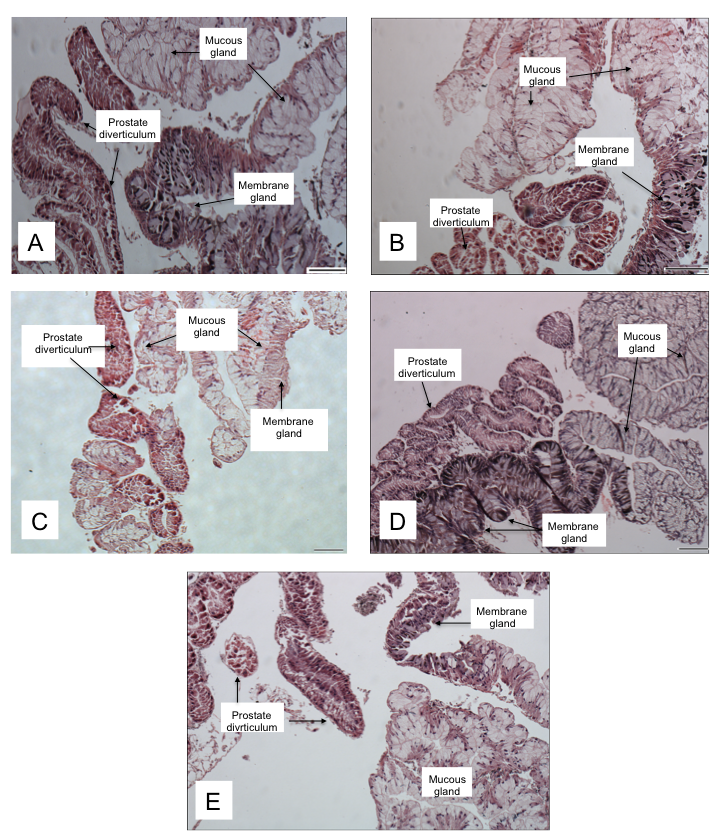

Supplement: S7 Fig — Representative photomicrographs; A) from dilution water control (DWC), B) solvent control (SC), C) 62.5 ng/L MT, D) 125 ng/L MT, and E) 500 ng/L MT (250ng/L treatment excluded due to high mortality). Scale bar 100 μm in each case. As seen in MT treatment, no significant treatment related effects to any of the three glands in the glandular complex were observed in DHT exposed snails compared to the control (either DWC or SC). (TIF) [file pone.0159852.s007.tif]

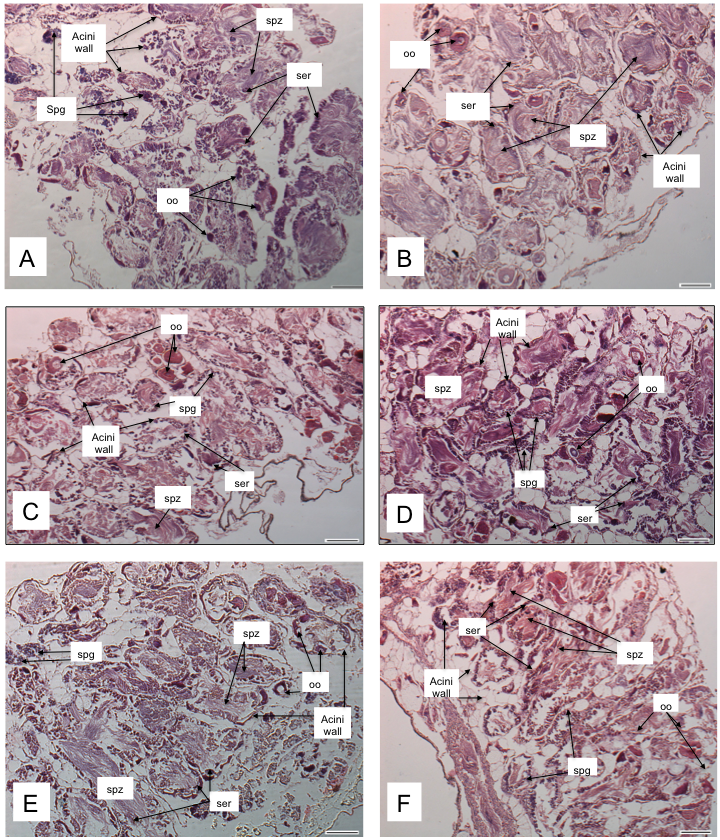

Supplement: S8 Fig — Representative photomicrographs; A) from dilution water control (DWC), B) solvent control (SC), C) 62.5 ng/L MT, D) 125 ng/L MT, E) 250 ng/L MT and F) 500 ng/L MT. Oo: Oocyte; Ser: Sertoli cell; Spg: Spermatogonia; Spd: Spermatid; Spz: Spermatozoa. (TIF) [file pone.0159852.s008.tif]

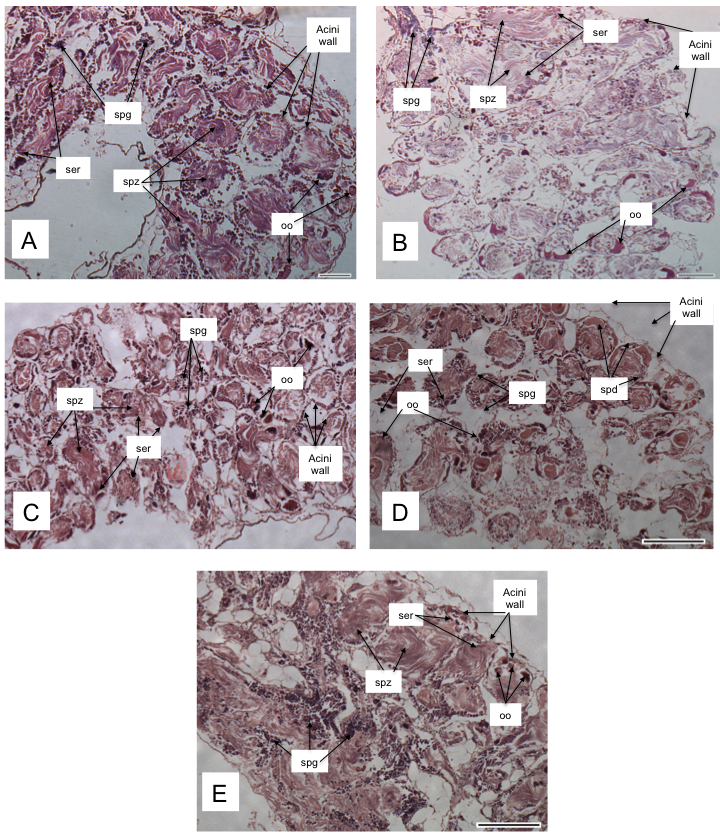

Supplement: S9 Fig — Representative photomicrographs; A) from dilution water control (DWC), B) solvent control (SC), C) 62.5 ng/L MT, D) 125 ng/L MT, and E) 500 ng/L MT. (250ng/L treatment excluded due to high mortality) Oo: Oocyte; Ser: Sertoli cell; Spg: Spermatogonia; Spd: Spermatid; Spz: Spermatozoa. (TIF) [file pone.0159852.s009.tif]
