# Supplementary material for: Steroid Androgen Exposure during Development Has No Effect on Reproductive Physiology of Biomphalaria glabrata
Source: PLoS One. 2016 Jul 22;11(7):e0159852. doi: 10.1371/journal.pone.0159852 (PMC4957768; doi:10.1371/journal.pone.0159852)
Supplement: S1 File — (PDF) [file pone.0159852.s010.pdf]

Table A

Descriptives per tank MT experiment

		N	Mean	Std. Deviation	Std. Error	95% Confidence Interval for Mean Lower Bound	95% Confidence Interval for Mean Upper Bound	Minimum	Maximum	Levene's P=	t-test for Equality of Means Sig. (2-tailed) P=	t-test for Equality of Means Mean Difference	t-test for Equality of Means Std. Error Difference	95% Confidence Interval of the Lower	95% Confidence Interval of the Upper
shell diameter (mm)	DWC-1	19	11.52	0.98	0.22	11.05	11.99	8.79	13.43	0.44	0.001*	1.28	0.35	0.57	2.00
	DWC-2	20	10.23	1.20	0.27	9.67	10.79	7.62	12.79						
	SC-1	17	11.55	2.37	0.58	10.33	12.77	5.67	15.03	0.759	<0.001*	3.96	0.75	2.44	5.48
	SC-2	21	7.59	2.24	0.49	6.57	8.61	3.57	10.61						
	62.5-1	16	9.69	2.46	0.61	8.38	11.00	4.97	14.07	0.163	0.149	1.18	0.79	-0.45	2.81
	62.5-2	14	8.51	1.78	0.48	7.48	9.54	5.46	11.49						
	125-1	19	10.90	1.83	0.42	10.02	11.78	6.67	13.48	0.314	<0.001*	2.49	0.58	1.30	3.68
	125-2	14	8.41	1.39	0.37	7.60	9.21	5.30	11.19						
	250-1	8	5.79	0.98	0.34	4.97	6.60	4.17	6.93	0.128	0.001	-2.62	0.66	-3.98	-1.27
	250-2	17	8.41	1.71	0.42	7.53	9.29	4.51	10.97						
	500-1	18	8.32	2.76	0.65	6.95	9.70	2.97	12.43	0.315	0.105	-1.70	1.01	-3.77	0.38
	500-2	10	10.02	2.14	0.68	8.49	11.55	6.55	13.51						
	Total	193													
total weight (g)	DWC-1	19	0.36	0.07	0.02	0.33	0.39	.16	.49	0.648	0.02*	0.05	0.02	0.01	0.10
	DWC-2	20	0.31	0.07	0.01	0.28	0.34	.18	.42						
	SC-1	17	0.42	0.17	0.04	0.33	0.50	.05	.69	0.216	<0.001*	0.24	0.04	0.14	0.33
	SC-2	21	0.18	0.11	0.02	0.13	0.23	.02	.38						
	62.5-1	16	0.27	0.14	0.03	0.20	0.34	.05	.51	0.103	0.14	0.06	0.04	-0.02	0.15
	62.5-2	14	0.21	0.09	0.02	0.15	0.26	.08	.36						
	125-1	19	0.32	0.11	0.03	0.27	0.37	.09	.46	0.242	0.001*	0.13	0.03	0.06	0.20
	125-2	14	0.19	0.08	0.02	0.14	0.24	.06	.34						
	250-1	8	0.07	0.02	0.01	0.05	0.09	.04	.10	0.044	<0.001*#	-0.12	0.02	-0.17	-0.07
	250-2	17	0.19	0.09	0.02	0.14	0.24	.04	.42						
	500-1	18	0.21	0.12	0.03	0.15	0.27	.03	.40	0.778	0.118	-0.07	0.05	-0.17	0.02
	500-2	10	0.28	0.11	0.04	0.20	0.36	.12	.47						
	Total	193													

# significant variance found; Welch's t Test used (equal variance not assumed)

* significant difference found between duplicate tanks of the same treatment

Table B

Descriptives per treatment MT experiment

		N	Mean	Std. Deviation	Std. Error	Interval for Mean Lower Bound	Interval for Mean Upper Bound	Minimum	Maximum
shell diameter (mm)	DWC	39	10.86	1.26	.20	10.45	11.27	7.62	13.43
	SC	38	9.36	3.02	.49	8.37	10.36	3.57	15.03
	62.5	30	9.14	2.22	.40	8.31	9.97	4.97	14.07
	125	33	9.84	2.06	.36	9.11	10.57	5.30	13.48
	250	25	7.57	1.95	.39	6.77	8.38	4.17	10.97
	500	28	8.93	2.65	.50	7.90	9.96	2.97	13.51
	Total	193							
total weight (g)	DWC	39	.33	.07	.01	.31	.35	.16	.49
	SC	38	.29	.18	.03	.23	.35	.02	.69
	62.5	30	.24	.12	.02	.20	.28	.05	.51
	125	33	.26	.12	.02	.22	.30	.06	.46
	250	25	.15	.10	.02	.11	.19	.04	.42
	500	28	.24	.12	.02	.19	.28	.03	.47
	Total	193							

Table C

shell diameter - MT

Tukey HSD

tank	N	Subset for alpha = 0.05 1	2	3	4
250-1	8	5.7888			
SC-2	21	7.5910	7.5910		
500-1	18		8.3239	8.3239	
125-2	14		8.4057	8.4057	
250-2	17		8.4100	8.4100	
62.5-2	14		8.5114	8.5114	
62.5-1	16		9.6906	9.6906	9.6906
500-2	10			10.0200	10.0200
DWC-2	20			10.2325	10.2325
125-1	19				10.8953
DWC-1	19				11.5174
SC-1	17				11.5512
		.320	.128	.238	.273

Means for groups in homogeneous subsets are displayed.

a. Uses Harmonic Mean Sample Size = 14.880

b. The group sizes are unequal. The harmonic mean of the group sizes is used. Type I error levels are not garenteed

Table D

Total weight - MT

Tukey HSD

tank1	N	Subset for alpha = 0.05 1	2	3	4	5
250-1	8	.0729				
SC-2	21	.1829	.1829			
125-2	14	.1886	.1886			
250-2	17	.1902	.1902			
62.5-2	14		.2051	.2051		
500-1	18		.2093	.2093		
62.5-1	16		.2701	.2701	.2701	
500-2	10		.2823	.2823	.2823	
DWC-2	20		0.3062	.3062	.3062	0.3062
125-1	19			0.3183	.3183	0.3183
DWC-1	19				.3582	0.3582
SC-1	17					0.4186
		.107	.071	.141	.495	0.147

Means for groups in homogeneous subsets are displayed.

a. Uses Harmonic Mean Sample Size = 12.271.

b. The group sizes are unequal. The harmonic mean of the group sizes is used. Type I error levels are not guaranteed.

Table E

Descriptives per tank

normalised ovotestis weight

	N	Mean	Std. Deviation	Std. Error	95% Confidence Interval for Mean Lower Bound	Upper Bound	Minimum	Maximum	Levene's P=	t-test for Equality of Means Sig. (2-tailed) P=	Mean Difference	Std. Error Difference	95% Confidence Interval of the Difference Lower	Upper
DWC-1	17	.024	.007	.002	.020	.028	.010	.040	0.878	0.846	0.001	0.003	-0.005	0.006
DWC-2	17	.023	.008	.002	.019	.028	.010	.050						
SC-1	13	.020	.006	.002	.016	.024	.010	.040	0.297	0.023*	0.007	0.003	0.001	0.013
SC-2	10	.013	.008	.002	.007	.018	.000	.030						
62.5-1	12	.022	.012	.004	.014	.030	.010	.040	0.698	0.469	-0.004	0.005	-0.015	0.007
62.5-2	11	.026	.013	.004	.017	.034	.010	.050						
125-1	18	.022	.007	.002	.019	.025	.010	.040	0.732	0.603	-0.002	0.003	-0.008	0.005
125-2	8	.024	.008	.003	.017	.031	.010	.040						
250-1	0													
250-2	11	.020	.008	.003	.015	.026	.010	.030						
500-1	13	.018	.008	.002	.014	.023	.010	.030	0.518	0.026*	-0.010	0.004	-0.019	-0.001
500-2	9	.028	.012	.004	.019	.038	.020	.060						
Total	139													

# significant variance found; Welch's t Test used (equal variance not assumed)

* significant difference found between duplicate tanks of the same treatment

Table F

Descriptives per treatment

normalised ovotestis weight

	N	Mean	Std. Deviation	Std. Error	Interval for Mean Lower Bound	Upper Bound	Minimum	Maximum
DWC	34	.0237	.00764	.00131	.0210	.0264	.01	.05
SC	23	.0167	.00764	.00159	.0134	.0200	.00	.04
62.5	23	.0238	.01236	.00258	.0184	.0291	.01	.05
125	26	.0226	.00704	.00138	.0197	.0254	.01	.04
250	11	.0202	.00844	.00254	.0146	.0259	.01	.03
500	22	.0222	.01076	.00229	.0174	.0270	.01	.06
Total	139							

Table G

ovo

Tukey HSD

tank	N	Subset for alpha = 0.05 1	2
SC-2	10	.0127	
500-1	13	.0181	.0181
SC-1	13	.0198	.0198
250-2	11	.0202	.0202
62.5-1	12	.0219	.0219
125-1	18	.0221	.0221
DWC-2	17	.0234	.0234
125-2	8	.0237	.0237
DWC-1	17	.0240	.0240
62.5-2	11		.0258
500-2	9		.0282
Sig.		.084	.178

Means for groups in homogeneous subsets are

b. The group sizes are unequal.

The harmonic mean of the group sizes is used.

Type I error levels are not guaranteed.

Table H

Descriptives per tank

normalised albumen weight

	N	Mean	Std. Deviation	Std. Error	95% Confidence Interval for Mean Lower Bound	95% Confidence Interval for Mean Upper Bound	Minimum	Maximum	Levene's P=	t-test for Equality of Means Sig. (2-tailed) P=	t-test for Equality of Means Mean Difference	t-test for Equality of Means Std. Error Difference	95% Confidence Interval of the Difference Lower	95% Confidence Interval of the Difference Upper
DWC-1	18	.0232	.00565	.00133	.0204	.0260	.020	.030	0.344					
DWC-2	20	.0258	.00792	.00177	.0221	.0295	.010	.040		0.263	-0.00257	0.00226	-0.00714	0.00201
SC-1	11	.0291	.01079	.00325	.0218	.0363	.020	.050	0.362					
SC-2	12	.0250	.01330	.00384	.0166	.0335	.010	.050		0.435	0.00405	0.00508	-0.00652	0.01461
62.5-1	11	.0299	.01738	.00524	.0182	.0415	.000	.060	0.312					
62.5-2	12	.0443	.01292	.00373	.0361	.0525	.030	.070		0.034*	-0.01444	0.00635	-0.02764	-0.00123
125-1	18	.0260	.00889	.00210	.0216	.0304	.010	.040	0.064					
125-2	9	.0393	.02364	.00788	.0212	.0575	.010	.090		0.042*	-0.01334	0.00623	-0.02617	-0.0052
250-1	2	.0300	0.02373	0.01678	-0.1832	0.2432	.010	.050	0.44					
250-2	12	.0258	0.01496	0.00432	0.0163	0.0353	.010	.050		0.734	0.004	0.012	-0.0222	0.03064
500-1	12	.0233	.01274	.00368	.0152	.0314	.010	.040	0.763					
500-2	9	.0311	.01119	.00373	.0225	.0397	.020	.050		0.159	-0.00782	0.00534	-0.019	0.00336
Total	146													

* significant difference found between duplicate tanks of the same treatment

Table I

Descriptives per treatment

normalised albumen weight

	N	Mean	Std. Deviation	Std. Error	Interval for Mean Lower Bound	Interval for Mean Upper Bound	Minimum	Maximum
DWC	38	.0246	.00697	.00113	.0223	.0269	.010	.040
SC	23	.0270	.01207	.00252	.0217	.0322	.010	.050
62.5	23	.0374	.01659	.00346	.0302	.0446	.000	.070
125	27	.0304	.01627	.00313	.0240	.0369	.010	.090
250	14	.0264	.01533	.00410	.0176	.0353	.010	.050
500	21	.0266	.01246	.00272	.0210	.0323	.010	.050
Total	146							

Table J

albumen

Tukey HSD

tank	N	Subset for alpha = 0.05 1	Subset for alpha = 0.05 2
DWC-1	18	.0232	
500-1	12	.0233	
SC-2	12	.0250	.0250
DWC-1	20	.0258	.0258
250-2	12	.0258	.0258
125-1	18	.0260	.0260
SC-1	11	.0291	.0291
62.5-1	11	.0299	.0299
250-1	2	.0300	.0300
500-2	9	.0311	.0311
125-2	9	.0393	.0393
62.5-2	12		.0443
Sig.		.266	.079

Means for groups in homogeneous subsets are

a. Uses Harmonic Mean Sample Size = 8.581.

b. The group sizes are unequal. The harmonic

Table K

Descriptives per tank

normalised gladular complex

	N	Mean	Std. Deviation	Std. Error	95% Confidence Interval for Mean Lower Bound	95% Confidence Interval for Mean Upper Bound	Minimum	Maximum	Levene's P=	t-test for Equality of Means Sig. (2-tailed) P=	t-test for Equality of Means Mean Difference	t-test for Equality of Means Std. Error Difference	95% Confidence Interval of the Difference Lower	95% Confidence Interval of the Difference Upper
DWC-1	12	0.2682	0.19495	0.05628	0.1443	0.3921	0.17	0.87						
DWC-2	14	0.2221	0.02429	0.00649	0.2081	0.2361	0.18	0.26	0.034	0.433#	0.04608	0.05665	-0.07821	0.17038
SC-1	7	0.1889	0.06688	0.02528	0.127	0.2507	0.13	0.33						
SC-2	4	0.246	0.06564	0.03282	0.1415	0.3504	0.18	0.32	0.665	0.204	-0.05711	0.04166	-0.15135	0.03713
62.5-1	9	0.2523	0.10898	0.03633	0.1686	0.3361	0.15	0.53						
62.5-2	9	0.3209	0.07852	0.02617	0.2606	0.3813	0.23	0.45	0.981	0.145	-0.06861	0.04477	-0.16352	0.02631
125-1	12	0.1987	0.0318	0.00918	0.1785	0.2189	0.14	0.25						
125-2	6	0.2315	0.06856	0.02799	0.1596	0.3035	0.16	0.35	0.074	0.177	-0.03283	0.02326	-0.08214	0.01648
250-1	0													
250-2	10	0.253	0.09922	0.03138	0.182	0.3239	0.12	0.42						
500-1	6	0.1927	0.02854	0.01165	0.1628	0.2227	0.15	0.23						
500-2	7	0.262	0.18378	0.06946	0.092	0.4319	0.15	0.67	0.98	0.383	-0.06924	0.07627	-0.2371	0.09863
Total	96													

significant variance found; Welch's t Test used (equal variance not assumed)

Table L

normalised gland complex

	N	Mean	Std. Deviation	Std. Error	Interval for Mean Lower Bound	Interval for Mean Upper Bound	Minimum	Maximum
DWC	26	.2434	.13258	.02600	.1898	.2969	.170	.870
SC	11	.2096	.06933	.02090	.1630	.2562	.130	.330
62.5	18	.2866	.09867	.02326	.2376	.3357	.150	.530
125	18	.2096	.04786	.01128	.1858	.2334	.140	.350
250	10	.2530	.09922	.03138	.1820	.3239	.120	.420
500	13	.2300	.13608	.03774	.1478	.3122	.150	.670
Total	96							

Levene Statistic P= 0.666

Table N

ANOVA

GC

	Sum of Squares	df	Mean Square	F	Sig.
Between Groups	.069	5	.014	1.241	.297
Within Groups	1.003	90	.011		
Total	1.072	95			

Table M

GC

Tukey HSD

tank	N	Subset for alpha = 0.05<br>1
SC-1	7	.1889
500-1	6	.1927
125-1	12	.1987
DWC-2	14	.2221
125-2	6	.2315
SC-2	4	.2460
62.5-1	9	.2523
250-2	10	.2530
500-2	7	.2620
DWC-1	12	.2682
62.5-2	9	.3209
Sig.		.340

Means for groups in homogeneous

a. Uses Harmonic Mean Sample

b. The group sizes are unequal. The
