# Supplementary material for: Steroid Androgen Exposure during Development Has No Effect on Reproductive Physiology of Biomphalaria glabrata
Source: PLoS One. 2016 Jul 22;11(7):e0159852. doi: 10.1371/journal.pone.0159852 (PMC4957768; doi:10.1371/journal.pone.0159852)
Supplement: S2 File — (PDF) [file pone.0159852.s011.pdf]

Table A

Descriptives per tank DHT experiment

		N	Mean	Std. Deviation	Std. Error	95% Confidence Interval for Mean Lower Bound	95% Confidence Interval for Mean Upper Bound	Minimum	Maximum	Levene's P=	t-test for Equality of Means Sig. (2-tailed) P=	t-test for Equality of Means Mean Difference	t-test for Equality of Means Std. Error Difference	95% Confidence Interval of the Lower	95% Confidence Interval of the Upper
shell diameter (mm)	DWC-1	15	11.65	1.62	0.42	10.76	12.55	8.27	14.39	0.616	0.126	-0.96	0.60	-2.20	0.29
	DWC-2	10	12.61	1.22	0.39	11.74	13.48	10.55	14.13						
	SC-1	20	11.26	1.17	0.26	10.71	11.80	7.61	12.84	0.482	0.314	0.40	0.39	-0.40	1.20
	SC-2	19	10.86	1.29	0.30	10.24	11.48	8.14	12.97						
	62.5-1	19	9.84	1.32	0.30	9.21	10.48	6.78	11.47	0.422	0.009*	-1.09	0.40	-1.89	-0.28
	62.5-2	19	10.93	1.11	0.25	10.40	11.46	7.70	12.77						
	125-1	15	12.52	1.57	0.41	11.65	13.39	9.41	15.17	0.806	0.271	-0.60	0.53	-1.69	0.49
	125-2	16	13.12	1.41	0.35	12.37	13.87	11.09	15.76						
	250-1	1	12.73					12.73	12.73						
	250-2	1	12.76					12.76	12.76						
	500-1	6	11.99	2.34	0.96	9.53	14.45	7.54	13.99	0.144	0.914	0.11	0.96	-2.01	2.22
	500-2	7	11.88	0.95	0.36	11.00	12.76	10.33	13.10						
	Total	148													
total weight (g)	DWC-1	15	0.36	0.12	0.03	0.29	0.42	.18	.60	0.473	0.169	-0.07	0.05	-0.16	0.03
	DWC-2	10	0.42	0.10	0.03	0.35	0.49	.26	.57						
	SC-1	20	0.37	0.08	0.02	0.33	0.41	.13	.53	0.346	0.287	0.03	0.03	-0.03	0.09
	SC-2	19	0.33	0.10	0.02	0.29	0.38	.13	.52						
	62.5-1	19	0.27	0.06	0.01	0.24	0.30	.11	.40	0.605	0.013*	-0.06	0.02	-0.10	-0.01
	62.5-2	19	0.33	0.07	0.02	0.29	0.36	.14	.47						
	125-1	15	0.46	0.13	0.03	0.39	0.54	.25	.67	0.475	0.667	-0.02	0.05	-0.11	0.07
	125-2	16	0.48	0.12	0.03	0.42	0.54	.33	.70						
	250-1	1	0.49					.49	.49						
	250-2	1	0.47					.47	.47						
	500-1	6	0.44	0.16	0.06	0.28	0.61	.15	.60	0.238	0.651	0.03	0.07	-0.11	0.17
	500-2	7	0.41	0.07	0.03	0.35	0.48	.30	.50						
	Total	148													

* significant difference found between duplicate tanks of the same treatment

Table B

Descriptives per treatment DHT experiment

		N	Mean	Std. Deviation	Std. Error	Interval for Mean Lower Bound	Interval for Mean Upper Bound	Minimum	Maximum
shell diameter (mm)	DWC	25	12.04	1.52	.30	11.41	12.66	8.27	14.39
	SC	39	11.06	1.23	.20	10.66	11.46	7.61	12.97
	62.5	38	10.39	1.32	.21	9.95	10.82	6.78	12.77
	125	31	12.83	1.49	.27	12.28	13.37	9.41	15.76
	250	2	12.75	0.02	.01	12.55	12.94	12.73	12.76
	500	13	11.93	1.66	.46	10.93	12.93	7.54	13.99
	Total	148							
total weight (g)	DWC	25	.38	.12	.02	.33	.43	.18	.60
	SC	39	.35	.09	.01	.32	.38	.13	.53
	62.5	38	.30	.07	.01	.28	.32	.11	.47
	125	31	.47	.12	.02	.43	.52	.25	.70
	250	2	.48	.01	.01	.35	.61	.47	.49
	500	13	.43	.11	.03	.36	.50	.15	.60
	Total	148							

Table C

shell diameter - DHT

Tukey HSD

tank1	N	Subset for alpha = 0.05		
		1	2	3
62.5-1	19	9.8437		
SC-2	19	10.8563	10.8563	
62.5-2	19	10.9300	10.9300	
SC-1	20	11.2575	11.2575	
DWC-1	15		11.6533	11.6533
500-2	7		11.8800	11.8800
500-1	6		11.9867	11.9867
125-1	15		12.5167	12.5167
DWC-2	10		12.6090	12.6090
125-2	16			13.1169
Sig.		.251	.058	.208

Means for groups in homogeneous subsets are displayed.

a. Uses Harmonic Mean Sample Size = 12.296.

b. The group sizes are unequal. The harmonic mean of the group sizes is used. Type I error levels are not garenteed

Table D

Total weight - DHT

Tukey HSD

tank1	N	Subset for alpha = 0.05			
		1	2	3	4
62.5-1	19	.2718			
62.5-2	19	.3289	.3289		
SC-2	19	.3346	.3346	.3346	
DWC-1	15	.3561	.3561	.3561	.3561
SC-1	20	.3668	.3668	.3668	.3668
500-2	7		.4141	.4141	.4141
DWC-2	10		.4225	.4225	.4225
500-1	6		.4445	.4445	.4445
125-1	15			.4625	.4625
125-2	16				.4821
Sig.		.377	.136	.063	.071

Means for groups in homogeneous subsets are displayed.

a. Uses Harmonic Mean Sample Size = 12.271.

b. The group sizes are unequal. The harmonic mean of the group sizes is used. Type I error levels are not guaranteed.

Table E

Descriptives per tank

normalised ovotestis weight

	N	Mean	Std. Deviation	Std. Error	95% Confidence Interval for Mean		Minimum	Maximum	Levene's P=	t-test for Equality of Means			95% Confidence Interval of the Difference	
					Lower Bound	Upper Bound				Sig. (2-tailed) P=	Mean Difference	Std. Error Difference	Lower	Upper
DWC-1	8	.022	.007	.002	.016	.028	.016	.038	0.452	0.372	-0.004	0.004	-0.013	0.005
DWC-2	9	.026	.010	.003	.018	.034	.016	.049						
SC-1	14	.022	.011	.003	.016	.029	.010	.050	0.487	0.855	0.001	0.004	-0.008	0.010
SC-2	9	.021	.008	.003	.016	.027	.010	.040						
62.5-1	15	.021	.005	.001	.018	.024	.011	.027	0.051	0.963	0.000	0.003	-0.005	0.005
62.5-2	15	.021	.008	.002	.016	.026	.009	.036						
125-1	12	.019	.006	.002	.015	.023	.008	.029	0.576	0.362	-0.002	0.002	-0.006	0.002
125-2	16	.021	.005	.001	.018	.023	.013	.030						
250-1	1	.003					.003	.003						
250-2	1	.022					.022	.022						
500-1	5	.019	.004	.002	.014	.024	.013	.024	0.037#	0.414	0.002	0.002	-0.003	0.007
500-2	7	.017	.002	.001	.015	.019	.013	.019						
Total	112													

significant variance found; Welch's t Test used (equal variance not assumed)

Descriptives per treatment

Table F

normalised ovotestis weight

	N	Mean	Std. Deviation	Std. Error	Interval for Mean		Minimum	Maximum
					Lower Bound	Upper Bound		
DWC	17	.0242	.00858	.00208	.0198	.0286	.02	.05
SC	23	.0218	.00986	.00206	.0176	.0261	.01	.05
62.5	30	.0211	.00680	.00124	.0186	.0236	.01	.04
125	28	.0200	.00542	.00102	.0179	.0221	.01	.03
250	2	.0122	.01323	.00936	-.1067	.1311	.00	.02
500	12	.0177	.00308	.00089	.0158	.0197	.01	.02
Total	112							

Levene Statistic P= 0.055

Table G

ANOVA

normalised ovotestis weight

	Sum of Squares	df	Mean Square	F	Sig.
Between Groups	.000	5	.000	1.841	.111
Within Groups	.006	106	.000		
Total	.006	111			

Table H

Descriptives per tank

normalised albumen weight

	N	Mean	Std. Deviation	Std. Error	95% Confidence Interval for Mean: Lower Bound	95% Confidence Interval for Mean: Upper Bound	Minimum	Maximum	Levene's P=	t-test for Equality of Means: Sig. (2-tailed) P=	t-test for Equality of Means: Mean Difference	t-test for Equality of Means: Std. Error Difference	95% Confidence Interval of the Difference: Lower	95% Confidence Interval of the Difference: Upper
DWC-1	15	.0199	.00697	.00180	.0160	.0237	.010	.030						
DWC-2	10	.0202	.00556	.00176	.0162	.0242	.010	.030	0.359	0.891	-0.00036	0.00263	-0.00582	0.00509
SC-1	19	.0303	.01180	.00271	.0246	.0360	.000	.050						
SC-2	16	.0315	.00951	.00238	.0264	.0366	.010	.050	0.323	0.748	-0.00119	0.00367	-0.00866	0.00628
62.5-1	18	.0263	.00757	.00178	.0225	.0300	.020	.040						
62.5-2	18	.0310	.01017	.00240	.0259	.0360	.020	.060	0.471	0.124	-0.00471	0.00299	-0.01079	0.00136
125-1	15	.0226	.00663	.00171	.0189	.0263	.010	.030						
125-2	16	.0328	.03595	.00899	.0137	.0520	.020	.160	0.099	0.124	-0.00471	0.00299	-0.01079	0.00136
250-1	1	.0260	.	.	.	.	.030	.030						
250-2	1	.0466	.	.	.	.	.050	.050						
500-1	6	.0244	.00686	.00280	.0172	.0316	.020	.030						
500-2	7	.0221	.00568	.00215	.0168	.0273	.020	.030	0.320	0.523	0.00229	0.00347	-0.00535	0.00994
Total	142													

Table I

Descriptives per treatment

normalised albumen weight

	N	Mean	Std. Deviation	Std. Error	Interval for Mean: Lower Bound	Interval for Mean: Upper Bound	Minimum	Maximum
DWC	25	.0200	.00632	.00126	.0174	.0226	.007	.034
SC	35	.0309	.01068	.00180	.0272	.0345	.003	.053
62.5	36	.0286	.00915	.00153	.0255	.0317	.016	.056
125	31	.0279	.02634	.00473	.0182	.0375	.012	.164
250	2	.0363	.01454	.01028	-.0943	.1669	.026	.047
500	13	.0232	.00610	.00169	.0195	.0268	.017	.035
Total	142							

Levene Statistic P= 0.290

Table J

ANOVA

normalised albumen weight

	Sum of Squares	df	Mean Square	F	Sig.
Between Groups	.002	5	.000	2.074	.072
Within Groups	.029	136	.000		
Total	.031	141			

Table K

Descriptives per tank

normalised gladular complex

	N	Mean	Std. Deviation	Std. Error	95% Confidence Interval for Mean Lower Bound	95% Confidence Interval for Mean Upper Bound	Minimum	Maximum	Levene's P=	t-test for Equality of Means Sig. (2-tailed) P=	t-test for Equality of Means Mean Difference	t-test for Equality of Means Std. Error Difference	95% Confidence Interval of the Difference Lower	95% Confidence Interval of the Difference Upper
DWC-1	9	.2543	.09642	.03214	.1802	.3284	.15	.45						
DWC-2	9	.1843	.02378	.00793	.1661	.2026	.14	.21	0.024#	0.064	0.06993	0.0331	-0.00499	0.14485
SC-1	13	.1810	.07450	.02066	.1359	.2260	.05	.30						
SC-2	12	.2282	.05438	.01570	.1936	.2627	.16	.36	0.246	0.085	-0.04724	0.02628	-0.1016	0.00713
62.5-1	14	.2360	.04619	.01235	.2094	.2627	.19	.32						
62.5-2	10	.2276	.06131	.01939	.1837	.2715	.16	.36	0.595	0.704	0.00843	0.0219	-0.037	0.05386
125-1	9	.1895	.06408	.02136	.1402	.2387	.11	.30						
125-2	4	.1947	.08712	.04356	.0561	.3333	.11	.31	0.577	0.905	-0.00524	0.04273	-0.09929	0.08881
250-1	0													
250-2	0													
500-1	5	.2033	.03663	.01638	.1578	.2487	.14	.23						
500-2	3	.2324	.05131	.02962	.1049	.3598	.18	.28	0.595	0.38	-0.02911	0.03074	-0.10433	0.04612
Total	88													

significant variance found; Welch's t Test used (equal variance not assumed)

Table L

Descriptives per treatment

normalised glandular complex

	N	Mean	Std. Deviation	Std. Error	Interval for Mean Lower Bound	Interval for Mean Upper Bound	Minimum	Maximum
DWC	18	.2193	.07704	.01816	.1810	.2576	.14	.45
SC	25	.2036	.06863	.01373	.1753	.2320	.05	.36
62.5ng	24	.2325	.05191	.01060	.2106	.2544	.16	.36
125ng	13	.1911	.06813	.01890	.1499	.2323	.11	.31
500ng	8	.2142	.04178	.01477	.1792	.2491	.14	.28
Total	88							

Levene Statistic P= 0.636

Table M

ANOVA

normalised gladular complex

	Sum of Squares	df	Mean Square	F	Sig.
Between Groups	.018	4	.005	1.101	.362
Within Groups	.344	83	.004		
Total	.362	87			
